# Endothelial extracellular vesicles enhance vascular self-assembly in engineered human cardiac tissues

**DOI:** 10.1088/1758-5090/ad76d9

**Published:** 2024-09-18

**Authors:** Karl T Wagner, Rick X Z Lu, Shira Landau, Sarah A Shawky, Yimu Zhao, David F Bodenstein, Luis Felipe Jiménez Vargas, Richard Jiang, Sargol Okhovatian, Ying Wang, Chuan Liu, Daniel Vosoughi, Dakota Gustafson, Jason E Fish, Carolyn L Cummins, Milica Radisic

**Affiliations:** 1 Institute of Biomedical Engineering, University of Toronto, 27 King’s College Circle, Toronto, ON M5S 1A1, Canada; 2 Department of Chemical Engineering and Applied Chemistry, University of Toronto, 27 King’s College Circle, Toronto, ON M5S 1A1, Canada; 3 Department of Pharmaceutical Sciences, Leslie Dan Faculty of Pharmacy, University of Toronto, 144 College St., Toronto, ON M5S 3M2, Canada; 4 Acceleration Consortium, University of Toronto, Toronto, ON, M5S 1A1, Canada; 5 Toronto General Hospital Research Institute, University Health Network, 200 Elizabeth Street, Toronto, ON M5G 2C4, Canada; 6 Department of Pharmacology and Toxicology, University of Toronto, Toronto, ON M5G 2C8, Canada; 7 Latner Thoracic Laboratories, Toronto General Hospital Research Institute, University Health Network, Toronto, Ontario M5G 2C4, Canada; 8 Institute of Medical Science, University of Toronto, Toronto, Ontario M5S 1A8, Canada; 9 Department of Laboratory Medicine and Pathobiology, University of Toronto, 27 King’s College Circle, Toronto, ON M5S 1A1, Canada; 10 Peter Munk Cardiac Centre, Toronto General Hospital,University Health Network, Toronto, Ontario M5G 2C4, Canada; 11 Terrence Donnelly Centre for Cellular & Biomolecular Research, University of Toronto, 27 King’s College Circle, Toronto, ON M5S 1A1, Canada

**Keywords:** vascularization, heart-on-a-chip, cardiac, extracellular vesicles, cardiac tissue engineering

## Abstract

The fabrication of complex and stable vasculature in engineered cardiac tissues represents a significant hurdle towards building physiologically relevant models of the heart. Here, we implemented a 3D model of cardiac vasculogenesis, incorporating endothelial cells (EC), stromal cells, and human induced pluripotent stem cell (iPSC)-derived cardiomyocytes (CM) in a fibrin hydrogel. The presence of CMs disrupted vessel formation in 3D tissues, resulting in the upregulation of endothelial activation markers and altered extracellular vesicle (EV) signaling in engineered tissues as determined by the proteomic analysis of culture supernatant. miRNA sequencing of CM- and EC-secreted EVs highlighted key EV-miRNAs that were postulated to play differing roles in cardiac vasculogenesis, including the let-7 family and miR-126-3p in EC-EVs. In the absence of CMs, the supplementation of CM-EVs to EC monolayers attenuated EC migration and proliferation and resulted in shorter and more discontinuous self-assembling vessels when applied to 3D vascular tissues. In contrast, supplementation of EC-EVs to the tissue culture media of 3D vascularized cardiac tissues mitigated some of the deleterious effects of CMs on vascular self-assembly, enhancing the average length and continuity of vessel tubes that formed in the presence of CMs. Direct transfection validated the effects of the key EC-EV miRNAs let-7b-5p and miR-126-3p in improving the maintenance of continuous vascular networks. EC-EV supplementation to biofabricated cardiac tissues and microfluidic devices resulted in tissue vascularization, illustrating the use of this approach in the engineering of enhanced, perfusable, microfluidic models of the myocardium.

## 1. Introduction

In cardiac tissue engineering and regenerative medicine, vascularization of engineered tissues presents a major challenge [1]. Bioengineered tissues have been vascularized previously through the incorporation of macroscale tubules (100+ mm in diameter), conduits, and networks within cell-laden hydrogels, fabricated via techniques such as 3D printing, soft lithography, and 3D stamping prior to endothelial cell (EC) coating of their intraluminal surfaces [2–6]. In vascular self-assembly, ECs seeded in hydrogels, often in the presence of supporting cells such as fibroblasts or mesenchymal stem cells (MSCs), are allowed to naturally migrate and organize into a patent and perfusable vessel network [3, 7]. Self-assembled vessels in such models form on the order of tens of microns in diameter, more akin to the size of native cardiac capillaries which typically measure up to ∼7 *µ*m in diameter [7, 8]. Although these platforms support the modeling of endothelial-myocardial barrier and crosstalk, many limitations remain such as unphysiologically low vascular density, insufficient cardiomyocyte-vessel proximity, as well as reduced long term stability and perfusability of the resulting vasculature [4, 5, 9, 10].

During development and into adulthood, multiple cell types in the heart co-develop in a generally healthy environment. This is not the case in tissue engineering; isolated, rounded cells are combined in different ratios and are often exposed to harsh enzymes. Specific concerns include anoikis, due to prolonged time in a rounded state without attachment, as well as apoptosis and necrosis due to ischemia. These and other factors can combine to disturb cell-circuit equilibria and cell-signaling, causing cells to behave and interact differently in engineered tissues than they would in a healthy developmental environment [11]. Understanding these effects is key to developing interventions to protect cells and enhance equilibria during tissue engineering and biofabrication, especially with respect to challenges in the vascularization of *in vitro* cardiac tissues.

Extracellular vesicles (EVs) represent a possible means for regulation of cell–cell signaling during cardiac vasculogenesis *in vitro*. Previous studies have already noted the critical roles that cardiac cell-secreted EVs play in maintaining healthy cardiac physiology, initiating disease progression when EV signaling becomes dysregulated, and mediating tissue repair and functional recovery when targeted EVs are applied in a therapeutic context [12, 13]. Their miRNA cargo has been a particular area of recent focus, as miRNAs enriched in EVs can regulate post-transcriptional gene expression by inducing instability or blocking translation of mRNA targets via the RNA interference pathway [12].

**Figure 1.**
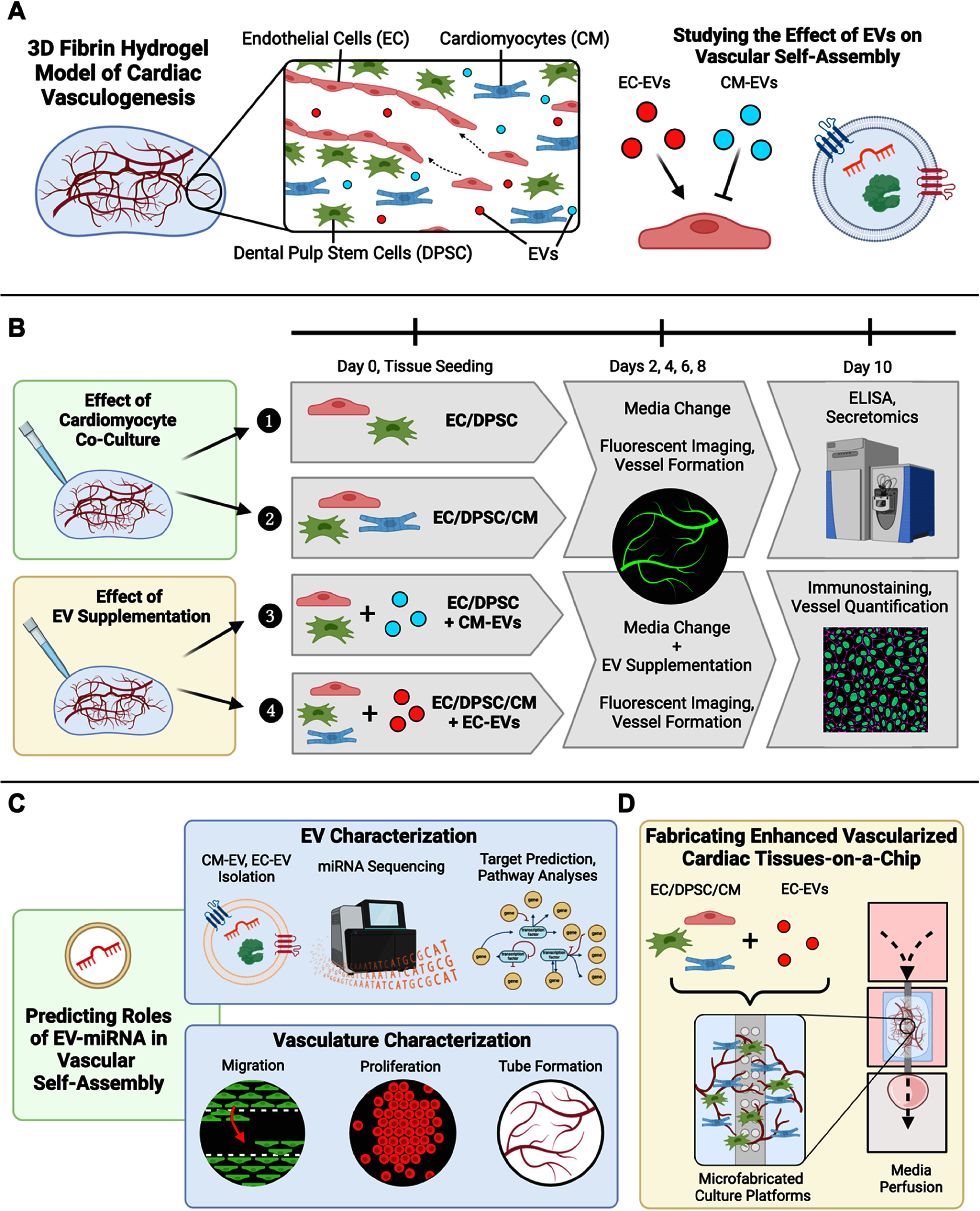
Overview of model system and experimental approach. (A) EV signaling between CMs and ECs was postulated to play a role in mediating vascular self-assembly in cardiac tissues *in vitro*. (B) Schematic diagram and timeline for the investigation of the role of cardiac EVs in microvascular self-assembly *in vitro*. The effect of CM co-culture on vascular self-assembly was first analyzed in 3D fibrin hydrogels in comparison to EC/DPSC hydrogels without CM. Next, the specific role of CM-EVs was examined by supplementing EC/DPSC cultures with isolated CM-EVs. Finally, the effects of supplemental EC-EVs on enhancing vascular self-assembly in EC/DPSC/CM co-cultures were tested. Tissues were imaged every two days to monitor vessel formation, followed by characterizations including ELISA and secretomic analysis of culture media as well as immunostaining analysis. (C) Isolation and characterization of EC- and CM-EVs included miRNA sequencing, miRNA target prediction, and downstream pathway analyses to predict potential biological processes modulated by EV miRNA. Highlighted pathways were paired with observed differences in EC migration, proliferation, and tube formation *in vitro* when vascular cells and tissues were supplemented with CM- or EC-EVs, together predicting potential roles of cardiac EVs on vascular self-assembly in engineered tissues. (D) The biological basis of enhanced cardiac vascularization demonstrated in 3D fibrin tissues was translated to microfabricated cardiac tissue-on-a-chip platforms to illustrate an approach to vascularize cardiac tissues in perfusable microfluidic devices and biofabricated cardiac tissues. Figure created using Biorender. EC: endothelial cell; DPSC: dental pulp stem cell; CM: cardiomyocyte; EV: extracellular vesicle; ELISA: enzyme-linked immunosorbent assay.

In this work, we utilized a 3D fibrin hydrogel model of vasculogenesis to study the effect of human iPSC-derived CMs on vascular self-assembly in engineered cardiac tissues (figure 1). We relied on enzyme-linked immunosorbent assay (ELISA) and proteomic analyses to identify key changes to the tissue secretome with the addition of CM to self-assembling vasculature. We analyzed vessel formation with and without CMs; examined the roles of EVs secreted by CMs or ECs, testing their effects on vascular self-assembly, and used miRNA sequencing and downstream pathway analyses to postulate the functional impacts of these EVs on cardiac vasculogenesis. By identifying aberrant EV signaling in the presence of CM-EVs during cardiac vasculogenesis *in vitro* and restoration of pro-vasculogenic signaling via EC-EV supplementation, we developed an approach to vascularize cardiac tissues in perfusable microfluidic devices and biofabricated cardiac tissues.

## 2. Results

### 2.1. Cardiomyocytes disrupt microvascular assembly in engineered cardiac tissues

Previous research has consistently demonstrated the necessity of pairing ECs with pericyte-like supporting cells to foster the formation of a stable vasculature *in vitro* [14]. Employing a fibrin hydrogel matrix, we co-cultured human umbilical vein endothelial cells (HUVECs) alongside human dental pulp stem cells (DPSCs). DPSCs were chosen for their enhanced efficacy in supporting vessel formation and stabilization compared to other stromal supporting cells like MSCs or fibroblasts [15–17]. Control tissues without CM, termed ‘EC/DPSC’, were seeded with equal numbers of GFP+ HUVEC and DPSC as supporting cells for vascular formation. In parallel, hydrogels containing the aforementioned cell mixture were used as a base with the addition of human iPSC-derived CM to create a cardiac vasculogenesis group, termed ‘EC/DPSC/CM’, at a cellular composition in line with previous studies shown to create functionally robust cardiac tissues [17, 18]. Representative flow cytometry analysis of iPSC-CM differentiation cultures indicated that a conservative estimate of 51.3% of differentiated cells were cTnT+ (supplemental figure S2(f)).

**Figure 2.**
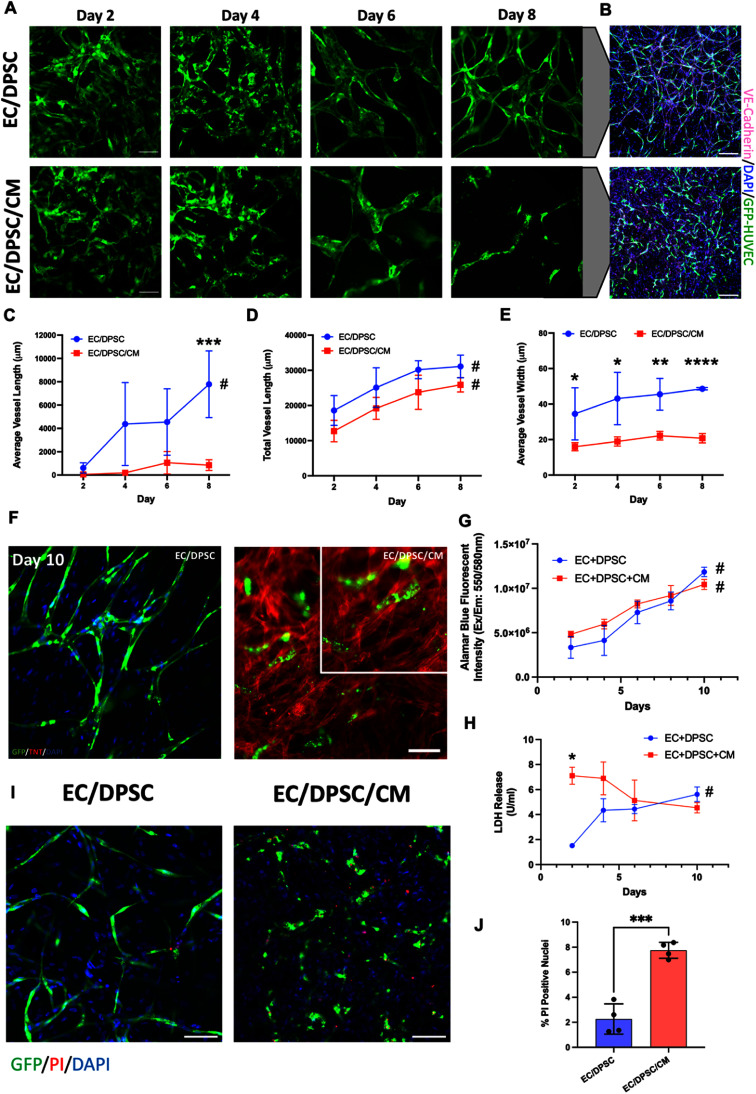
Co-culture of CM with EC and DPSC in a 3D fibrin hydrogel model of vasculogenesis causes disruption of vessel formation. (A) Representative fluorescent microscope images of GFP-HUVEC co-cultured with DPSC and iPSC-derived CM reveal differences in vessel morphology over 8 d of culture. Scale bar: 100 *μ*m. (B) VE-Cadherin immunostaining of tissues at their day 10 endpoint. Scale bar: 200 *μ*m. (C)‒(E) Comparative quantification of (C) average vessel length, (D) total vessel length, and (E) average vessel width over time in culture. *N* = 5 both groups. (F) Representative fluorescent images of cells stained with cardiac troponin T (red). Scale bar: 50 *μ*m. (G) Quantification of Alamar Blue metabolic activity in cell culture media. *N* = 3. (H) Quantification of LDH concentration in cell culture media. *N* = 3. (I) Representative fluorescent images of cells stained with propidium iodide (PI, red) on day 10. Scale bar: 100 *μ*m. (J) Quantification of % PI positive nuclei in fibrin tissues, day 10. *N* = 4 Statistical analysis performed by repeated measures ANOVA to test the effect of time within a group, and a Student t-test with post-hoc Holm–Šídák test to account for multiple comparisons to test specific hypothesized differences between the groups at singular timepoints. *Denotes significant difference between groups at a given timepoint; Student t-test with post-hoc Holm–Šídák test for multiple comparisons (where relevant), *P < 0.05, **P < 0.01, ***P < 0.001, ****P < 0.0001. For (C)‒(E), (G), (H): #Denotes significant change between day 2 and day 8 (C)‒(E) or day 2 and day 10 (G) and (H) within a given group, *P* < 0.05; repeated measures two-way ANOVA with Geisser–Greenhouse correction followed by Tukey’s multiple comparisons with individual variances computed for each comparison. Data presented as mean ± standard deviation (SD).

Qualitative assessment of EC vessel formation in tissues over time revealed a distinct reduction in organized vessel tubes in tissues containing CM as early as day 4 of culture, with increased gaps between cells and shorter vessels evident by day 8 when compared to EC/DPSC controls (figure 2(a)). VE-Cadherin staining further highlighted these discontinuities (figure 2(b)). Fluorescent image quantification revealed a significant reduction to average vessel length in EC/DPSC/CM tissues on day 8 compared to EC/DPSC alone (figure 2(c)). Average vessel length increased through 8 d of culture for EC/DPSC tissues (as denoted by ‘#’ in figure 2(c)) but stayed consistently low for EC/DPSC/CM tissues. Total vessel length was also slightly reduced at each timepoint in the presence of CM (figure 2(d)). The average width of self-assembling vessels was consistently lower in tissues containing CM compared to those without, with differences becoming more pronounced over time in culture. (figure 2(e), supplemental figure S1).

Cardiac troponin T (cTnT) staining highlighted the elongated structure of CM sarcomeres among the discontinuous vessels in EC/DPSC/CM tissues (figure 2(f)). We also performed Alamar blue analysis on tissues, finding that metabolic activity did not differ significantly between groups throughout the culture period (figure 2(g)). Though EC/DPSC/CM tissues were initially seeded with more cells and might be expected to have increased metabolic activity, this effect was balanced by the observation that lactate dehydrogenase (LDH) release, associated with cell death and a clinical marker of cardiac injury, was also significantly higher on day 2 in CM-laden hydrogels (figure 2(h)). No significant differences were observed on the days following this spike. Endpoint propidium iodide (PI) staining also revealed a significant increase in the presence of dead cells in EC/DPSC/CM tissues (figures 2(i) and (j)).

Digestion of fibrin tissues was performed after 8 days of culture for flow cytometry analysis of relative differences in cellular composition between groups (supplemental figure S2). Despite the same starting point, the final relative amount of ECs harvested from tissues on day 8 was almost 3.5 fold higher in EC/DPSC tissues compared to the EC/DPSC/CM group (supplemental figure S2(d)). Even with the differences in EC population and cell death, we observed that both groups stabilized to a similar equilibrium final cell concentration at the end of the culture period (supplemental figure S2(c)), with proliferation of DPSCs and non-myocytes from differentiation cultures accounting for similar metabolic activity towards the endpoint of culture.

To assess whether hypoxia was the cause of disrupted vessel formation within the core of thick 3D tissues containing metabolically demanding CMs, we seeded EC/DPSC and EC/DPSC/CM gels as a uniform thin layer coating the culture surface with an approximate thickness of 100 *µ*m, below the recognized oxygen diffusion limit for tissues *in vitro* and *in vivo* [19, 20]. Disrupted vessel formation in thin layers of gel containing CM mirrored that of 3D tissues (supplemental figure S3). We also seeded 3D EC/DPSC tissues at three times the original cell density to increase oxygen demand, as well as tissues containing a 1:5 ratio of ECs to DPSCs to test whether proportional dilution of the EC population drove impaired vessel formation. We visually observed successful and stable formation of vessels at each of the tested conditions (supplemental figure S4). Collectively, these results eliminated higher cell density (i.e. hypoxia) and EC dilution as a driving force for impaired vessel formation, pointing to the importance of cell type (i.e. CM presence).

### 2.2. The presence of cardiomyocytes upregulates endothelial activation markers and alters EV signaling in engineered tissues

**Figure 3.**
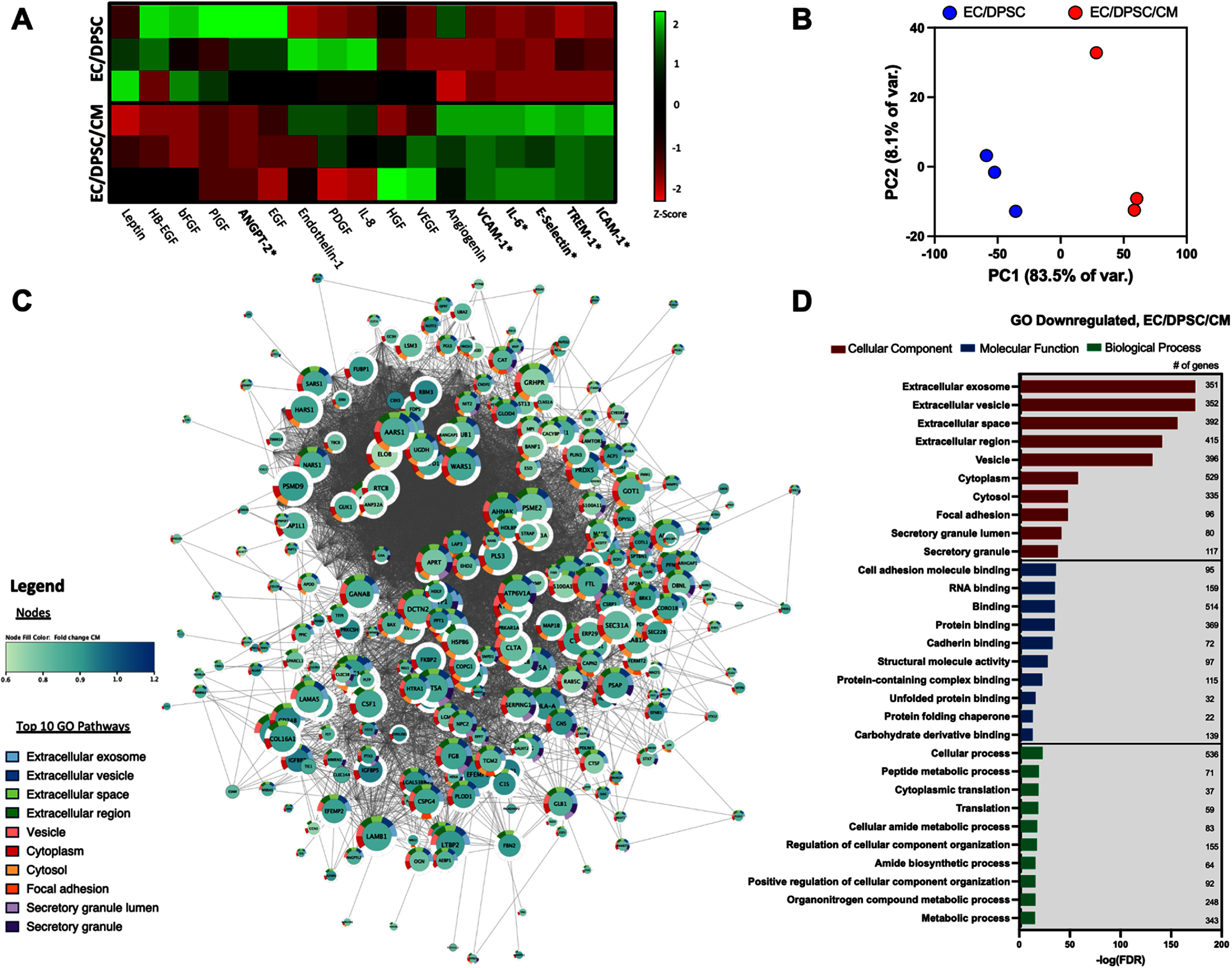
Cytokine and proteomic analyses of conditioned media revealed increased EC activation and modulation of EV-related gene ontology (GO) terms with the addition of CMs to vascular tissues. (A) Angiogenesis array and endothelial marker ELISA analysis of culture media revealed differing expression of key factors between EC/DPSC and EC/DPSC/CM. (B) Proteomic principal component analysis of the EC/DPSC vs. EC/DPSC/CM secretome. (C) Network diagram of proteins significantly downregulated in EC/DPSC/CM secretome when compared to EC/DPSC. Significance determined by false discovery rate adjusted *P*-value (FDR) <0.05 (D) Top 10 most significantly (sorted by smallest FDR) enriched GO cellular component (CC), molecular function (MF), and biological process (BP) terms for significantly downregulated proteins in EC/DPSC/CM.*P < 0.05, Student t-test with post-hoc Holm–Šídák test for multiple comparisons. *N* = 3 for all samples.

To investigate potential causes of CM-mediated disruption of vascular self-assembly, we performed secretome analyses on conditioned media collected from EC/DPSC and EC/DPSC/CM tissues at their endpoint, employing both global proteomic characterization as well as targeted validation of specific factors of interest using ELISA. ELISA analysis for a select panel of endothelial activation and inflammation markers [21–25] revealed significant upregulation of intercellular adhesion molecule-1 (ICAM-1), vascular cell adhesion molecule-1 (VCAM-1), E-selectin, triggering receptor expressed on myeloid cells-1 (TREM-1), and interleukin 6 (IL-6) in EC/DPSC/CM hydrogels compared to EC/DPSC (figure 3(a)). We also observed significant downregulation of angiopoietin-2 (ANGPT-2) in the EC/DPSC/CM group. Several other angiogenesis-related markers in figure 3(a) exhibited a slight but non-significant reduction in the EC/DPSC/CM group, with epidermal growth factor (EGF) (*P* = 0.054) and basic fibroblast growth factor (bFGF) (*P* = 0.088) falling just outside the threshold for significance (*P* < 0.05).

Global proteomic analysis of the secretome via liquid chromatography-mass spectrometry (LC-MS/MS) (figure 3(b), supplemental tables 1 and 2) revealed 577 proteins that were significantly downregulated in tissues containing CM (figure 3(c), supplemental video S1) and 19 that were significantly upregulated (supplemental figure S4), when compared to EC/DPSC controls. Gene ontology (GO) analysis on significantly downregulated proteins indicated that the top 10 enriched pathways ranked by significance (false discovery rate adjusted *P*-value, FDR) all came from the GO cellular component (CC) database (figure 3(d), supplemental table 3). Seven of the ten top enriched GO CC terms were associated with EVs, possessing strong FDR values ranging from 1.55 × 10^−174^–7.20 × 10^−39^ (figure 3(c)) and number of represented genes ranging from 80 to 415 (figure 3(d)). Of the secreted proteins upregulated in tissues containing CM, the cardiac-associated insulin-like growth factor 2 (IGF2) had the highest enrichment (supplemental figure S5, supplemental table 4) [26]. Only two GO CC terms were significantly enriched for upregulated genes: extracellular space and extracellular region; however, with fewer representative genes (12 and 14 respectively).

### 2.3. ECs and cardiomyocytes secrete EVs with differentially enriched miRNA

**Figure 4.**
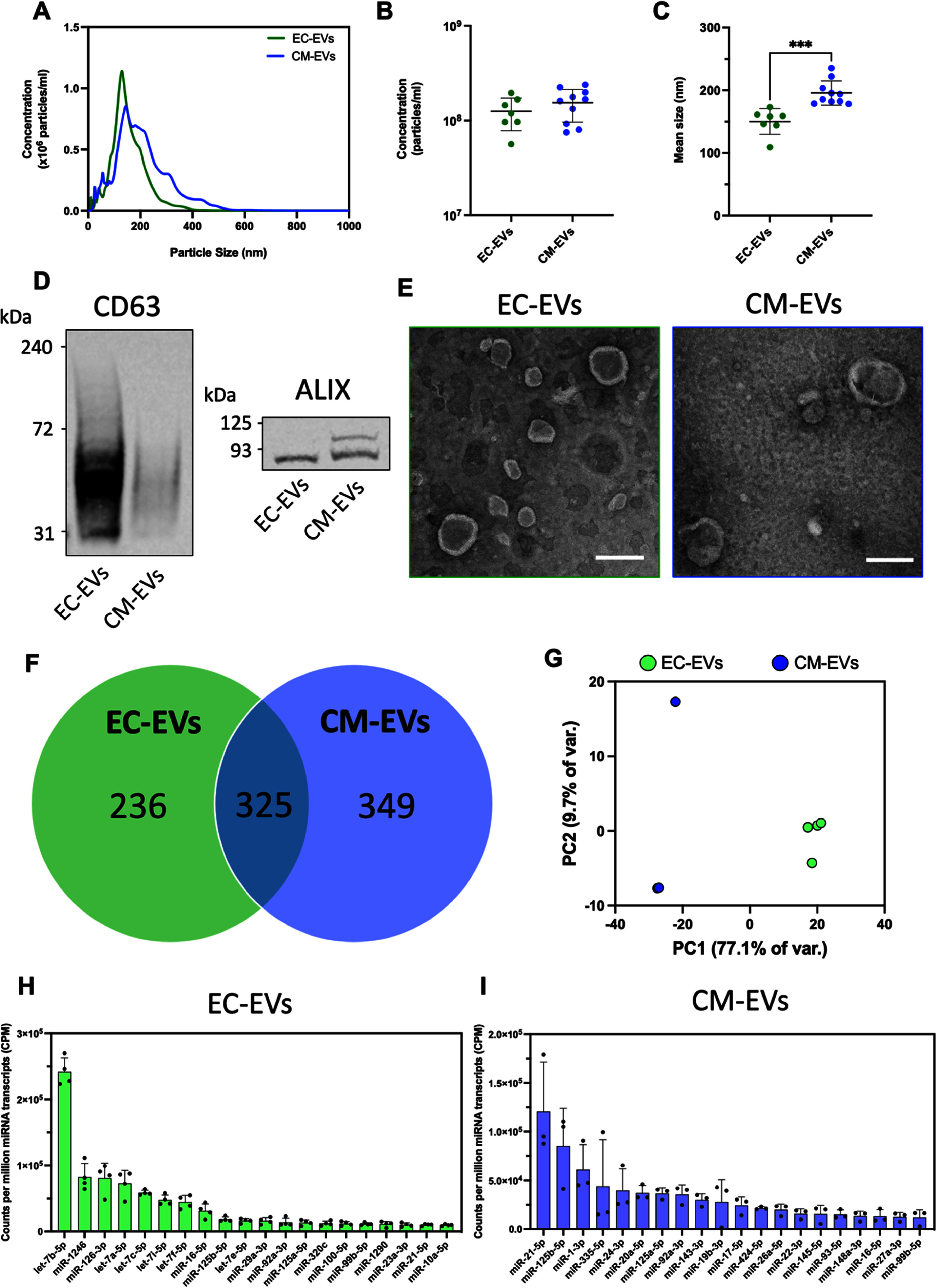
Comparative characterization of extracellular vesicles isolated from endothelial cells and cardiomyocytes. (A) Averaged size distribution curves, (B) concentration, and (C) mean size of isolated particles as detected by nanoparticle tracking analysis (NTA). *N* = 7 for EC-EVs, *N* = 10 for CM-EVs. (D) Western blot detection of CD63 and ALIX markers in EV isolates. (E) Transmission electron microscope (TEM) images of EVs. Scale bar = 200 nm. (F) miRNA sequencing detected numerous distinct and overlapping miRNA between EV types. (G) Principal component analysis of EV miRNA. *N* = 4 for EC-EVs, *N* = 3 for CM-EVs (H), (I) Top 10 most abundant miRNAs found in (H) HUVEC-EVs (*N* = 4) and (I) CM-EVs (*N* = 3). Welch’s t-test; ***P < 0.001. Data presented as mean ± SD.

Informed by our findings from secretome analyses which indicated significant changes to EV-related proteins in vascularized cardiac tissues, we next sought to isolate and characterize EVs secreted by ECs and CMs to assess their potential roles in mediating vascular self-assembly and to guide our targeted follow-up studies of their functional effects in engineered cardiac tissues. To enable us to characterize each type of EV independently, we isolated EVs from pure 2D monocultures of EC and CM separately via PEG-based precipitation from conditioned media. This approach ensured a clear presence of only one cell type and prevented possible interactions in 3D co-cultures that could confound correct EV identification. Nanoparticle tracking analysis (NTA) indicated similar size distribution profiles for both sources of EVs, with no significant differences in EV concentration but a slightly larger mean size of 196 nm for CM-EVs compared to 150 nm for HUVEC-EVs (figures 4(a)–(c)). Western blots confirmed the presence of the EV membrane marker CD63 and the intraluminal EV-associated protein ALIX in isolates (figure 4(d), supplemental figure S6), while transmission electron microscopy (TEM) allowed us to visualize isolated particles displaying a characteristic cup-shaped morphology (figure 4(e)) [12, 27]. miRNA sequencing of isolated EVs uncovered numerous distinct and overlapping miRNAs detected between the two cell sources (figure 4(f), supplemental table 5) and clear separation of the sample groups via principal component analysis (figure 4(g)). The top 20 most abundant miRNA detected in EC- and CM-EVs are depicted in figures 4(h) and (i) respectively. For CM-EVs, they included the cardiac associated miR-1-3p [12] as well as miR-21-5p and miR-125b-5p. For EC-EVs, these included 6 members of the let-7 family of miRNAs as well as miR-1246 and miR-126-3p.

Differential expression analysis of EV miRNA (supplemental figures S7(a) and S8(a); supplemental table 6), followed by plots of fold change versus miRNA abundance (supplemental figures S7(b) and (c)) highlighted miRNAs that were both abundant and upregulated for each EV type (figures 4(h) and (i); supplemental figures S7(a)–(c)). We selected a subset of key miRNAs for both EC-EVs and CM-EVs using a cut-off threshold of miRNAs that were both upregulated and within the top 20 most abundant for each respective EV type (blue text labels in supplemental figures S7(d) and (e)). miRNA target prediction generated lists of mRNA species potentially modulated by each EV type. Kyoto Encyclopedia of Genes and Genomes (KEGG) analysis of these targets identified biological pathways possibly affected by EV miRNAs (supplemental figure S9(a), supplemental table 7). Multiple pathways uniquely enriched for EC-EVs (denoted by ^, supplemental figure S9(a)) were correlated with one or more members of the let-7 family of miRNAs, which comprised 6 of the 11 key EC-EV miRNAs. Uniquely enriched pathways for CM-EVs (denoted by #, supplemental figure S9(a)) included the transforming growth factor- *β* (TGF-*β*) signaling pathway, associated with targets of miR-20a-5p, miR-145-5p, and miR-424-5p; and prion disease and renal cell carcinoma, associated with miR-148a-3p. Gene ontology biological process (GO BP) analysis using predicted mRNA targets (sorted by fold enrichment) revealed ‘coronary vasculature morphogenesis’ and ‘positive regulation of vascular EC proliferation’ in the top 10 significantly enriched terms for EC-EVs (supplemental figure S9(b), supplemental tables 8–11). For CM-EVs, top enriched GO BP terms included ‘(positive) regulation of EC chemotaxis’ (supplemental figure S9(c)). Comparing predicted CM-EV miRNA targets to proteins actually downregulated in EC/DPSC/CM secretome uncovered 95 overlapping genes which, when used as a subset for GO analysis, replicated 6 of the top 10 enriched GO terms from the secretome analysis in figure 3(d) (supplemental figures S8(b) and (c); supplemental table 12).

### 2.4. CM-EVs inhibit EC migration and proliferation, reducing vascular tube formation in 3D tissues *in vitro*

**Figure 5.**
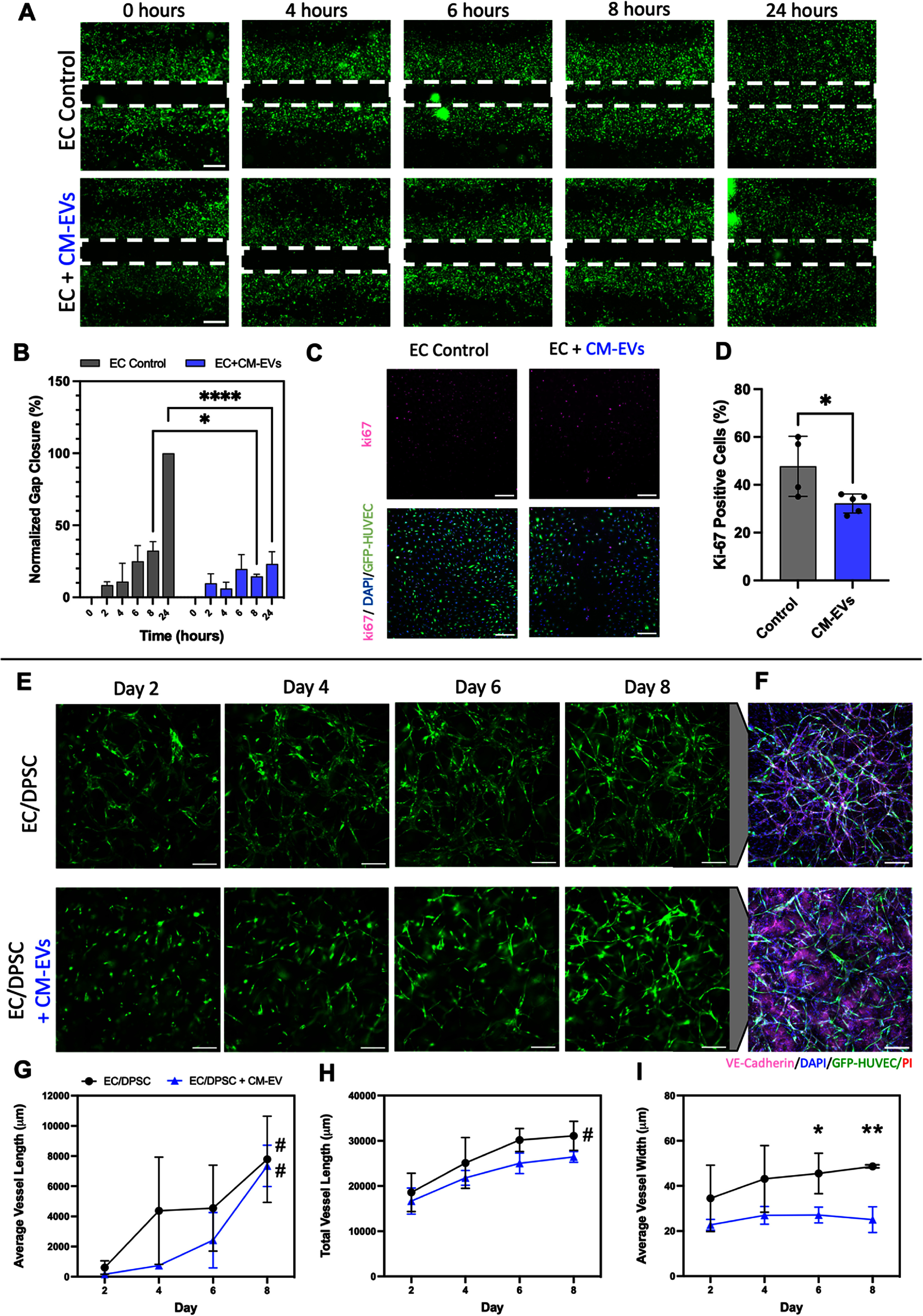
CM-EVs attenuate vessel formation *in vitro*. (A) Scratch-wound assay testing the effect of CM-EVs on EC migration. Scale bar: 500 *μ*m. (B) Quantification of gap closure from scratch-wound assay. *N* = 4 Control, *N* = 3 CM-EVs. (C) Ki-67 imaging determined the effect of CM-EVs on EC proliferation. Scale bar: 200 *μ*m. (D) Quantification of fraction of positive Ki-67 cells. *N* = 4 Control, *N* = 5 CM-EVs. (E) Representative fluorescent images of GFP-HUVECs in 3D EC/DPSC fibrin hydrogels depicting results of vascular self-assembly in the presence of CM-EVs. Scale bar: 200 *μ*m. (F) VE-Cadherin immunostaining of tissues at their day 10 endpoint. Scale bar: 200 *μ*m. (G)‒(I) Comparative quantification of (G) average vessel length, (H) total vessel length, and (I) average vessel width over time in culture. *N* = 5 EC/DPSC, *N* = 3 EC/DPSC + CM-EVs. Statistical analysis performed by repeated measures ANOVA to test the effect of time within a group, and a Student t-test with post-hoc Holm–Šídák test to account for multiple comparisons to test specific hypothesized differences between the groups at singular timepoints. *Denotes significant difference between groups at a given timepoint; Student t-test with post-hoc Holm–Šídák test for multiple comparisons (where applicable), *P < 0.05, **P < 0.01, ***P < 0.001, ****P < 0.0001. For (G)-(I): #Denotes significant change between day 2 and day 8 within a given group, *P* < 0.05; repeated measures two-way ANOVA with Geisser–Greenhouse correction followed by Tukey’s multiple comparisons with individual variances computed for each comparison. Data presented as mean ± SD.

Following our observations of CM-mediated disruption of vascular self-assembly and subsequent EV characterization and predictive pathway analyses that implicated a possible impact of CM-EVs on modulating EC migration, we next tested the physical impacts of CM-EVs on vascular cell and tissue cultures. Adapting a scratch-wound assay in a simple 2D HUVEC monolayer, we observed that the addition of isolated CM-EVs to EC culture media at a dose of ∼1.0 × 10^9^ EVs ml^−1^, consistent with previous studies, significantly reduced the rate of EC migration and gap closure over 24 h compared to control cultures not supplemented with CM-EVs (figures 5(a) and (b)) [28, 29]. Ki-67 staining of ECs after 24 h of CM-EV treatment indicated a reduced proportion of positive proliferating cells when compared to untreated controls (figures 5(c) and (d)).

To extend these observations to *in vitro* vascularization of 3D tissues, we seeded EC/DPSC in 3D fibrin hydrogels and supplemented their culture media with isolated CM-EVs throughout the culture period. We found that even in the absence of CM cells, CM-EV supplementation led to visible disruption of vascular tube formation when compared to untreated EC/DPSC controls (figure 5(e)). As soon as day 4 and through day 8, vasculature appeared more discontinuous and less organized in cultures supplemented with CM-EVs. Average vessel length increased from day 2 to day 8 for both groups (figure 5(g)), whereas total vessel length increased significantly from day 2 to day 8 only for the control group not supplemented with CM-EVs (figure 5(h)). Average vessel width was significantly lower on days 6 and 8 for tissues treated with CM-EVs, with such vessels appearing mostly as singular elongated ECs compared to the larger clusters of elongated cells that assembled into vessel networks in untreated control tissues (figure 5(i), supplemental figure S10(a)). Staining and fluorescent imaging of VE-Cadherin and GFP+ HUVEC at the endpoint of culture further emphasized the visual reduction in vascular organization and density with CM-EV supplementation (figure 5(f)).

### 2.5. EC-EV supplementation enhances vascular self-assembly in cardiac tissues *in vitro*

**Figure 6.**
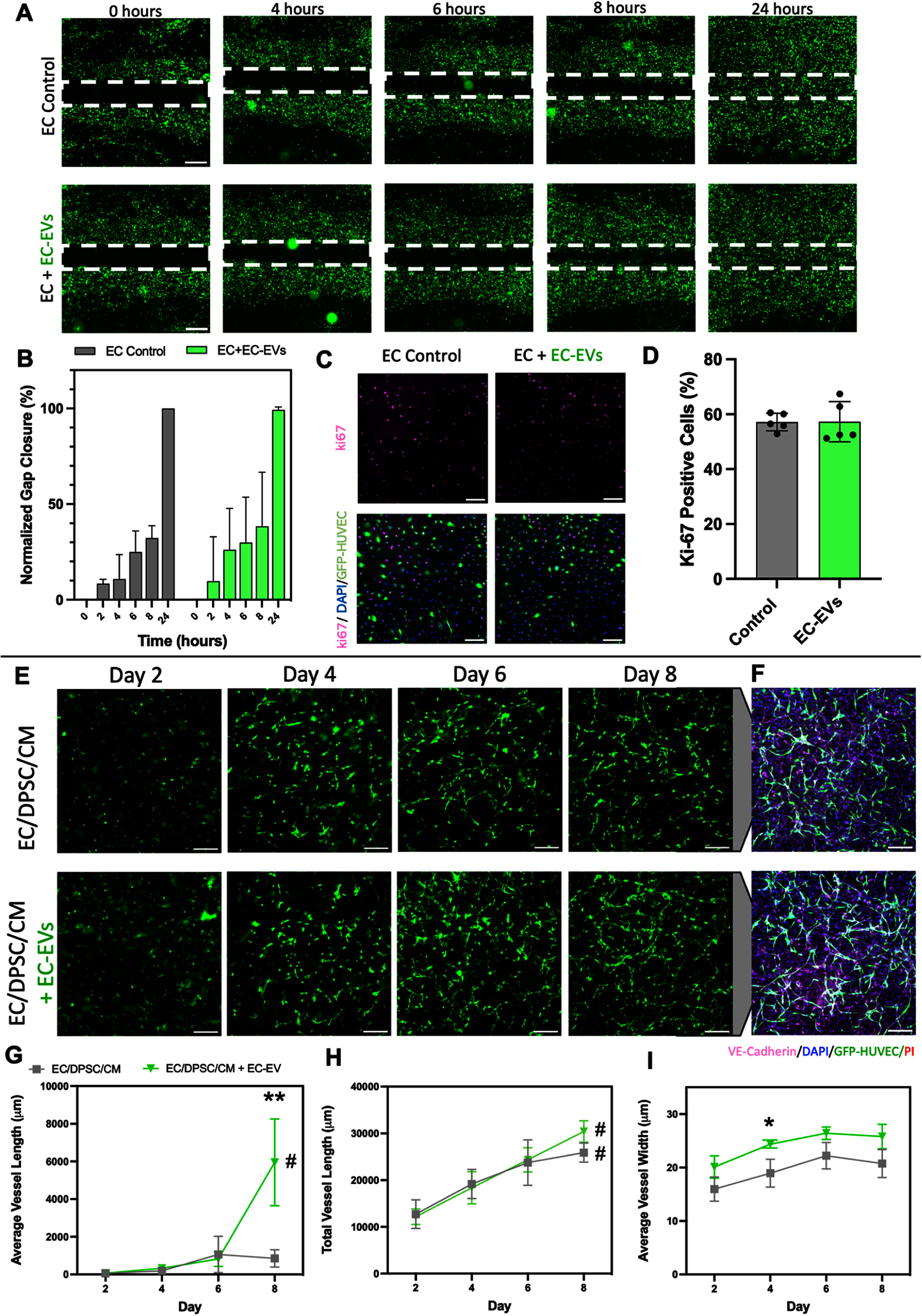
EC-EVs enhance vessel formation in engineered cardiac tissues. (A) Scratch-wound assay testing the effect of EC-EVs on EC migration. Scale bar: 500 *μ*m. (B) Quantification of gap closure from scratch-wound assay. N = 4 both groups. (C) Ki-67 imaging determined the effect of CM-EVs on EC proliferation. Scale bar: 200 *μ*m. (D) Quantification of fraction of positive Ki-67 cells. *N* = 5 both groups. (E) Representative fluorescent images of GFP-HUVECs in 3D EC/DPSC/CM fibrin hydrogels depicting results of vascular self-assembly in the presence of EC-EVs. Scale bar: 200 *μ*m. (F) VE-Cadherin immunostaining of tissues at their day 10 endpoint. Scale bar: 200 *μ*m. (G)‒(I) Comparative quantification of (G) average vessel length, (H) total vessel length, and (I) average vessel width over time in culture. *N* = 5 EC/DPSC/CM, *N* = 4 EC/DPSC/CM + EC-EVs. Statistical analysis performed by repeated measures ANOVA to test the effect of time within a group, and a Student t-test with post-hoc Holm–Šídák test to account for multiple comparisons to test specific hypothesized differences between the groups at singular timepoints. *Denotes significant difference between groups at a given timepoint; Student t-test with post-hoc Holm–Šídák test for multiple comparisons (where applicable), **P < 0.01. For (G)‒(I): #Denotes significant change between day 2 and day 8 within a given group, *P* < 0.05; repeated measures two-way ANOVA with Geisser–Greenhouse correction followed by Tukey’s multiple comparisons with individual variances computed for each comparison. Data presented as mean ± SD.

After observing the impacts of CM-EVs on vasculogenesis *in vitro*, we sought to test the effects of EC-EV supplementation to our vascularized cardiac cultures to see if we could improve vessel formation by engineering a more pro-vasculogenic EV signaling environment. Our predictive miRNA analyses (supplemental figures S7 and S9) postulated that EC-EVs could potentially modulate EC migration, vascular tube morphogenesis, and proliferation, prompting us to investigate these effects further *in vitro*. As with CM-EVs, we first tested the effects of EC-EVs in a 2D HUVEC scratch-wound assay, observing that a dose of ∼6.3 × 10^7^ EVs ml^−1^ of isolated EC-EVs added to culture media did not actually cause a significant difference in EC migration and gap closure compared to untreated controls over 24 h (figures 6(a) and (b)). Similarly, Ki-67 staining of these ECs also did not detect a significant difference in the proportion of positive proliferating cells at this dose (figures 6(c) and (d)).

To assess EC-EV impacts on vascular tube formation within cardiac tissues, we next seeded 3D EC/DPSC/CM cardiac tissues and added supplemental isolated EC-EVs to the media of a subset of tissues throughout the culture period. EC-EVs stained with lipophilic dye (DiI) were added to 2D HUVEC cultures to qualitatively confirm uptake of EVs via fluorescent imaging (supplemental figures S11(a) and (b)). We also added stained EC-EVs to 3D fibrin tissues for quantitative flow cytometry analysis of uptake, finding that 18.3% ± 1.2% of cells harvested from 3D fibrin tissues were positive for DiI-stained EC-EVs 24 h after supplementation (supplemental figures S11(c) and (d)). A correlation between lipid nanoparticle transfection of cells and uptake of EVs has previously been noted since both processes rely on common internalization and intracellular trafficking mechanisms [30]. Thus, based on the significantly higher lipofectamine transfection efficiency of DPSCs compared to the other cell types in our cardiac tissues (supplemental figure S13), it is likely that most of these DiI-positive cells were DPSCs.

We observed that the addition of EC-EVs to 3D tissues (∼1.3 × 10^8^ EVs ml^−1^) did lead to a noticeable visual improvement in vascular tube continuity in cardiac tissues at day 6 and day 8 when compared to untreated EC/DPSC/CM controls (figure 6(e)). Quantification revealed that average vessel length increased over time and was significantly higher on day 8 in EC-EV supplemented tissues compared to controls, in which individual vessels remained short and discontinuous throughout the culture period (figure 6(g)). Total vessel length increased from day 2 to day 8 for both groups, with no significant differences between groups (figure 6(h)). Average vessel width was significantly higher on day 4 for the EC-EV supplemented group compared to controls (figure 6(i), supplemental figure S10(b)). Staining and fluorescent imaging of VE-Cadherin and GFP+ HUVEC emphasized the improvements in vessel continuity and size that were evident with the addition of EC-EVs to cardiac tissue media (figure 6(f)).

**Figure 7.**
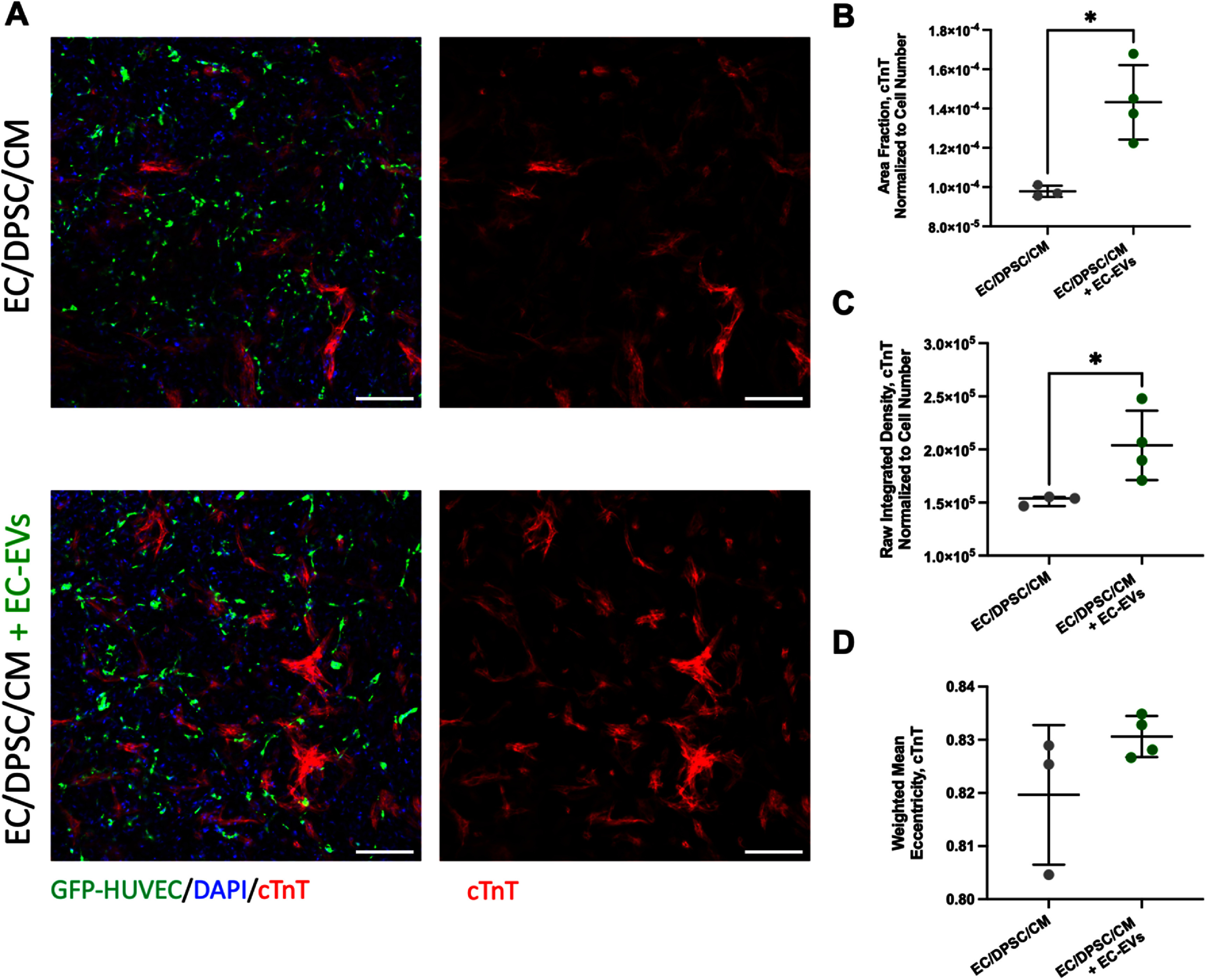
EC-EVs enhance cardiomyocyte coverage in vascularized cardiac tissues. (A) Representative immunostaining images of cardiac troponin T (cTnT) highlighted the morphology of cardiomyocytes in EC/DPSC/CM tissues alongside those supplemented with EC-EVs. Day 10, scale bars: 200 *μ*m. (B)‒(D) Quantification of (B) normalized area fraction, (C) normalized integrated density, and (D) weighted mean eccentricity of cTnT immunostaining. Statistical analysis was performed by student t-tests (*P* < 0.05 considered significant). *N* = 3 for EC/DPSC/CM, *N* = 4 for EC/DPSC/CM + EC-EVs. Data presented as mean ± standard deviation (SD).

Flow cytometry of day 8 fibrin tissues did not reveal significant differences in relative cellular populations between tissues with and without EC-EV supplementation (supplemental figure S12). Tissues were also stained for cTnT to assess CM structure at the culture endpoint (figure 7(a)). Tissues supplemented with EC-EVs exhibited significant improvements in cTnT fractional area coverage (figure 7(b)) and integrated density (figure 7(c)) compared to those not treated with EC-EVs, with no significant difference in CM elongation (as assessed through cTnT eccentricity) (figure 7(d)).

### 2.6. Transfection with let-7b-5p or miR-126-3p enhances vascular self-assembly in cardiac tissues

**Figure 8.**
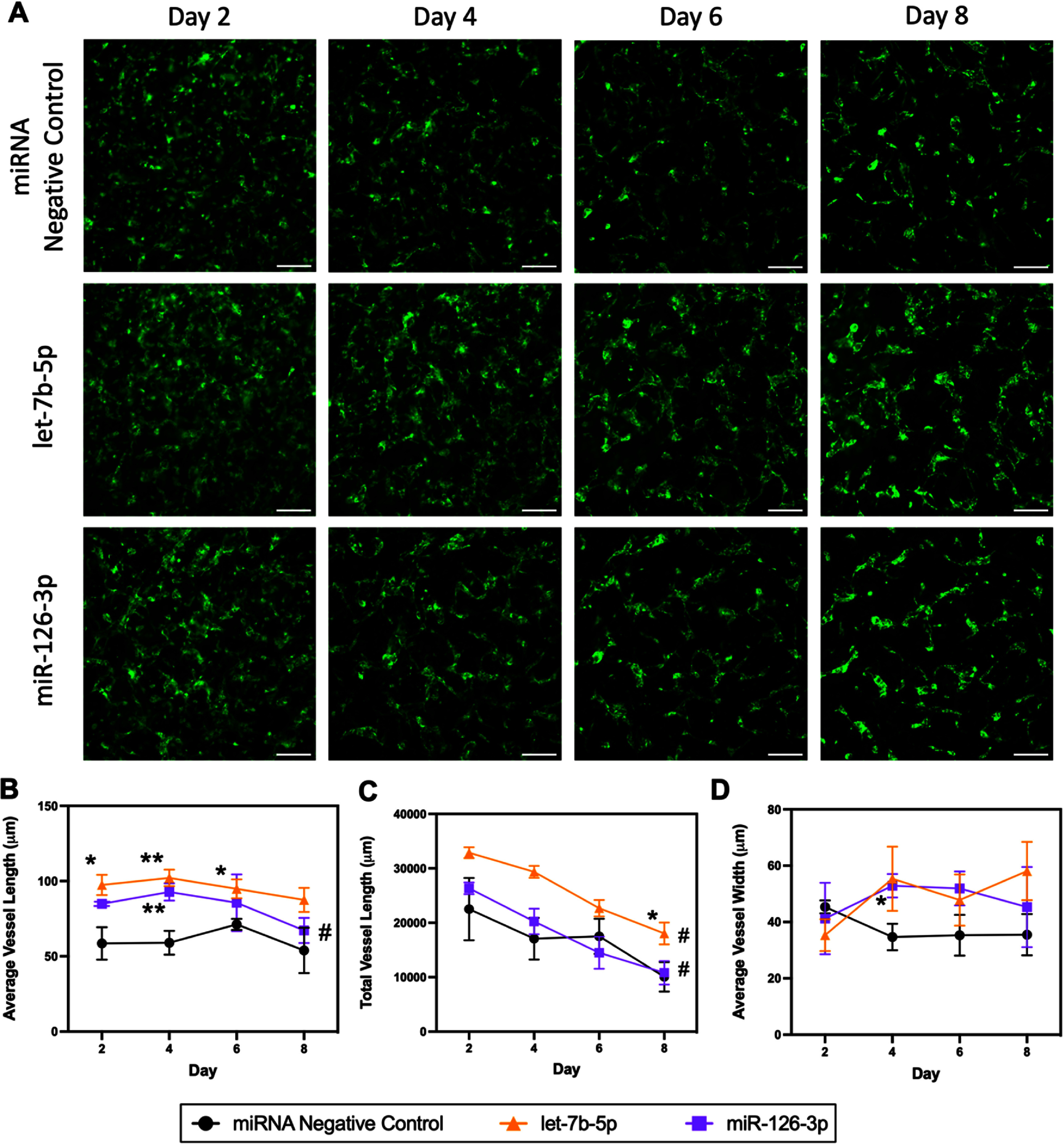
Transfection of cells with selected key EC-EV miRNAs before fibrin hydrogel seeding enhances vessel formation and stability in 3D cardiac tissues. (A) Representative fluorescent images of GFP-HUVECs in 3D EC/DPSC/CM fibrin hydrogels depicting results of vascular self-assembly where cells were pre-transfected with either negative control miRNA, let-7b-5p, or miR-126-3p before tissue seeding. Scale bars: 200 *μ*m. (B)‒(D) Comparative quantification of (B) average vessel length, (C) total vessel length, and (D) average vessel width over time in culture. *N* = 4 all groups. Statistical analysis performed by repeated measures ANOVA (to test the effect of time within a group), and a Student t-test with post-hoc Holm–Šídák test to account for multiple comparisons (to test specific hypothesized differences between the groups at singular timepoints). *Denotes significant difference between a key miRNA (let-7b-5p or miR-126-3p, purple or orange symbols) and the miRNA negative control group (black symbols) at a given timepoint; Student t-test with post-hoc Holm–Šídák test for multiple comparisons, *P < 0.05, **P < 0.01. #Denotes significant change between day 2 and day 8 within a given group, *P* < 0.05; repeated measures two-way ANOVA with Geisser–Greenhouse correction followed by Tukey’s multiple comparisons with individual variances computed for each comparison. Data presented as mean ± standard deviation (SD).

To assess the *in vitro* impacts of specific miRNAs on vascular self-assembly in engineered cardiac tissues, we selected two key EC-EV miRNAs highlighted in our predictive analyses for transfection tests: let-7b-5p (the most abundant member of the let-7 family in EC-EVs) and miR-126-3p (figure 4(h); supplemental figure S7(d)). We separately performed lipofectamine-based transfection of 2D cultures of ECs, DPSCs, and CMs with one of the two selected miRNAs, or a miRNA negative control mimic, for 24 h prior to seeding 3D fibrin tissues with transfected cells and monitoring vascular self-assembly through serial fluorescent imaging (figure 8(a)). 2D transfection efficiency was also estimated for each cell type via transfection with fluorescent siRNA, indicating that approximately 99% of DPSCs internalized lipofectamine-siRNA complexes, compared to 26% and 6% for CMs and ECs respectively (supplemental figure S13). Tissues fabricated with let-7b-5p transfected cells exhibited significantly increased average vessel length on days 2, 4, and 6 as well as a significantly higher total vessel length on day 8 compared to the negative control miRNA mimic group (figures 8(b)–(d)). Tissues fabricated with miR-126-3p transfected cells exhibited significantly higher average vessel length and vessel width on day 4 compared to negative controls (figures 8(b)–(d)). These results confirmed that the beneficial effects of EC-EVs on vasculogenesis in cardiac tissues could be exerted through key species in their miRNA cargo.

### 2.7. EC-EV supplementation in microfluidic devices and biofabricated cardiac tissues enables vascularization

**Figure 9.**
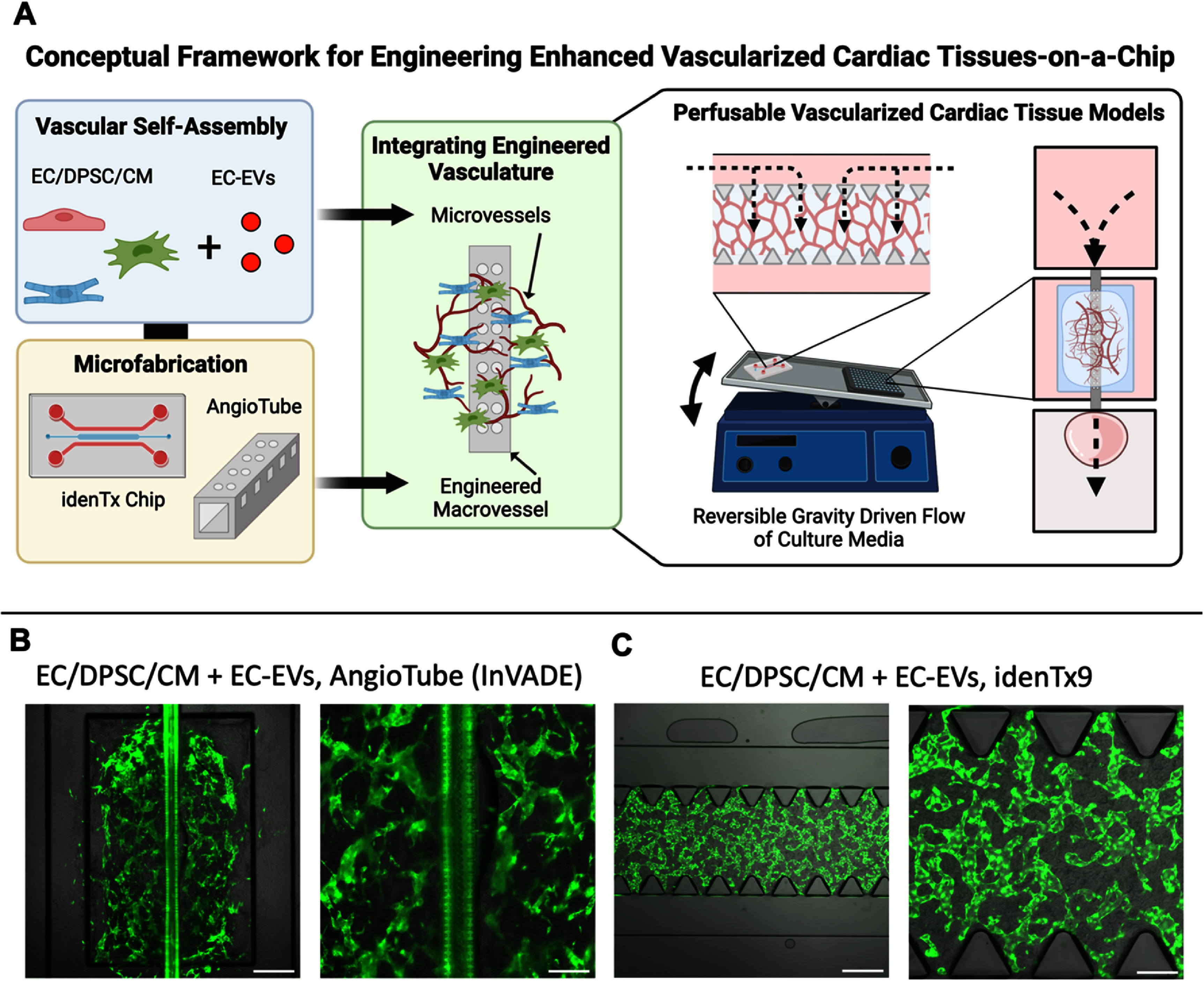
Biofabricated cardiac tissues and organ-on-a-chip platforms show robust vascularization in the presence of EC-EVs. (A) Schematic outlining the conceptual framework for integrating EC-EV supplementation of cardiac cultures with microfabricated platforms. (B) Representative images of a vascularized, EC-EV supplemented tissue cultured on the InVADE platform, depicting a tissue surrounding a microfabricated, perfusable AngioTube. Day 6, scale bars: 500 *µ*m and 200 *µ*m, respectively. (C) Representative images of a vascularized, EC-EV supplemented tissue cultured on the idenTx9 microfluidic platform. Day 2, scale bars: 500 *µ*m and 200 *µ*m, respectively.

Applying the same biological basis of EC-EV supplemented cardiac tissues already established in 3D fibrin hydrogels, we sought to highlight their broader applicability for vascularization of biofabricated cardiac tissues (figure 9(a)). The biofabricated InVADE culture platform represents one such system, consisting of a microfabricated polymer ‘Angiotube’ with integrated microholes and reversible gravity-driven flow of culture media through the 100 *µ*m lumen to replicate vessel perfusion *in vivo* [5, 6]. We biofabricated InVADE culture plates then seeded cardiac tissues around the outside of the Angiotube lumen. Applying the same fibrin tissue seeding protocol and EC-EV supplementation regimen established prior, we engineered vascularized cardiac tissues that integrated our self-assembled microvessels around the Angiotube ‘macrovessel’ lumen perfused via gravity driven flow (figure 9(b)). As a further example, we adapted our EC-EV supplemented fibrin tissues for seeding in the commercially available idenTx9 chip (AIM Biotech), creating cardiac tissues with self-assembled vascular networks integrated within a perfusable microfluidic chip (figure 9(c)) [31].

## 3. Discussion

To develop physiologically relevant models of *in vitro* human cardiac tissue for drug screening, disease modeling, or implantation and *in vivo* integration, it is imperative to enhance the fidelity and stability of vasculature in engineered cardiac constructs. Though the extra cells in CM-laden tissues could add physical impediments to vessel formation, we questioned whether CM-secreted factors further contributed to inhibited vasculogenesis. EC retention was reduced and cell death was initially elevated in CM-laden tissues. Similar trends in vessel formation within thin layers of hydrogel and gels with increased cell densities discounted the possibility that hypoxia or proportional dilution of the EC population were primary factors in vessel disruption. These observations motivated us to look to the secretome of CM-containing tissues for clues to explaining the increasingly apparent deficiencies that we noted in cardiac vasculature over time.

While targeted ELISA analysis noted that EC activation and inflammation markers increased in the presence of CM, proteomics and gene ontology analysis provided a broader view of secretome differences, with seven of the top ten most significantly downregulated GO terms for EC/DPSC/CM related to EV signaling. This trend motivated us to narrow our focus to investigating the specific roles of EV signaling in mediating cardiac vascularization *in vitro*, though we note that significant changes to other processes, including cell adhesion molecular binding, warrant further mechanistic investigations in future studies beyond the scope of this work. Recognizing the contextual complexities of the EV signaling environment existing in engineered cardiac tissues, we hypothesized that if EVs play a critical role in mediating vessel formation then dysregulation of the EV signaling environment with the addition of CMs could lead to a disruption of the vasculogenic balance in tissues.

Several previous studies have suggested that CM-EVs can possess anti-angiogenic properties [32, 33]; others have shown pro-angiogenic effects, highlighting the importance of context [33, 34]. Though the effects of CM-EVs on vascular disruption were not as dramatic as the effects of CM cells themselves, it was evident from our *in vitro* studies of CM-EV supplementation that CM-EVs have the capacity to contribute to impeded vasculogenesis in engineered tissues. Secondly, we identified EC-EVs as a potential supplement for cardiac cultures to enhance vascular self-assembly and pro-vasculogenic signaling in tissues.

ECs themselves are also known to be key to regulating healthy cardiac physiology, mediating disease progression, participating in reparative responses in the heart *in vivo* [35, 36], and enhancing the maturation of stem-cell derived CM [37]. EC-EVs are a dominant player in the vasculogenic EV environment in the absence of CM, and some previous studies have observed enhanced vascular tube formation with the application of EC-EVs *in vitro* [38, 39]. Despite numerous existing studies that have analyzed EC-EV-mediated angiogenesis in ECs, fewer have examined the role of EC-EVs in cardiac vasculogenesis [38–43]. Related work has tested supplemental EC-EVs in cardiac tissue-on-a-chip cultures as a prophylactic for reducing ischemic injury [44].

Since our findings from transfection efficiency screening suggest that a significant proportion of supplemental EVs may have been internalized by DPSCs and not directly by ECs, it is possible that our observations of enhanced vascularization could be mediated by EC-EVs reinforcing a more pro-vasculogenic phenotype in DPSCs. We demonstrated a similar mechanism previously through a combination of single cell sequencing and secretomics in cardiac tissues incorporating primitive macrophages [17]. Specifically, primitive macrophages in co-culture acted on DPSCs to enhance their pro-vasculogenic signaling and thereby stabilize the vasculature in cardiac tissues *in vitro* [17].

In an effort to further understand the mechanism behind EC-EV effects, we determined that most KEGG pathways uniquely enriched for EC-EV miRNAs led back to the let-7 family, which comprised over half of our key EC-EV miRNAs. Let-7 miRNAs are known to have diverse functions and sometimes conflicting effects in vascular development, homeostasis, barrier function, and pathology [45]. They have strong anti-proliferative effects on ECs during development, but can still display positive effects on EC migration or barrier function *in vitro* and *in vivo* [46]. In one study, let-7-5p overexpression in ECs resulted in inflammation and EC activation [47] while in another, let-7a-5p impaired EC migration and tube formation via targeting transforming growth factor-*β* receptor 3 (TGFBR3) [48]. A different study that tested let-7a-5p and let-7b-5p found that each species applied separately to cells improved EC viability and sprout formation, yet they negatively affected EC metabolism and viability with co-application [49]. Contradictory findings have been linked to a potential dose-dependency [38]. Let-7b-5p in particular was shown to directly target caspase 3 (CASP3) in HUVECs to enhance pro-survival signaling. The same study also validated the effects of let-7b-5p on decreasing the expression of StAR-related lipid transfer domain protein 13 (STARD13) and frizzled class receptor 4 (FZD4), genes with previously noted roles in negative and biphasic regulation of angiogenesis, respectively [49]. Notably, we also identified these three genes as predicted targets of all six key let-7 miRNAs included in our bioinformatic analysis of EC-EV cargo. We suspect that maintaining a balance of let-7 miRNAs could be imperative to healthy vessel development and stability. Restoring this balance in the context of enhanced vessel formation in cardiac tissues may require small enough doses to minimize anti-proliferative effects while maximizing potential improvements to EC integrity and organization imparted by the let-7 family. miR-126-3p, enriched in our EC-EVs, has also shown utility in promoting EC tube formation, potentially through supressing sprouty related EVH1 domain containing 1 (SPRED1) [42]. SPRED1 is an inhibitor of the pro-angiogenic extracellular signal-regulated kinase 1/2 (ERK1/2) pathway and another predicted target identified in our EC-EV miRNA analysis [42]. The fact that ‘coronary vascular morphogenesis’ and ‘positive regulation of vascular EC proliferation’ were among the top 10 enriched GO biological process terms for EC-EV miRNA targets solidified their potential to modulate the balance of cardiac vasculogenesis *in vitro*.

EC-EVs supported enhanced vascular continuity in cardiac constructs, a property which is key to building functional and perfusable vascular networks. This result, along with validation of the positive effects of the key EC-EV miRNAs let-7b-5p and miR-126-3p through direct transfection, reflected the predictive analyses and literature that suggested a role of EC-EVs in beneficially modulating vessel morphology and vessel maintenance. Thus, in this context, we were able to harness EC-EVs to shift the balance of pro-vasculogenic signaling that was initially disrupted by CMs and CM-EVs, enhancing vessel self-assembly in engineered cardiac tissues. Finally, we extended the same findings in fibrin hydrogels to applications in more complex biofabricated models of *in vitro* heart tissue. We showed proof-of-concept regarding how EC-EVs can be harnessed to support microvascularization within biofabricated cardiac tissue-on-a-chip platforms to engineer complex and physiologically relevant *in vitro* models of the heart incorporating microfluidic perfusion. The benefits observed with EC-EV supplementation highlight the concept that studying and then selectively manipulating the EV milieu can allow us to favourably moderate the fine balance of vasculogenesis in engineered tissues.

Besides EV-miRNA, numerous other key signaling factors have been noted for their importance in modulating vessel formation *in vitro* and *in vivo*. Factors such as vascular endothelial growh factor (VEGF) and TGF-*β* can have biphasic roles in the process of vasculogenesis, either promoting or inhibiting vessel formation and endothelial barrier function depending on the cells and tissues involved, physiological context, and synergistic interactions with other environmental factors [38, 50–53]. VEGF in particular illustrates this concept, playing important known roles in promoting EC migration and angiogenesis yet failing in several clinical trials of VEGF and VEGF-gene therapy due to creation of leaky vasculature or angioma formation [25, 54–57]. Ultimately, it is a fine balance of many different processes that determines success in tissue vascularization, driving a continued need to find alternative methods and interventions to enhance pro-vasculogenic signaling and create stable vasculature in engineered cardiac tissues [2].

## 4. Limitations and future work

The compositional profile of biomolecular cargo packaged into secreted EVs depends on factors including source cell type, the physiological state of the parent cell, environmental cues, and interactions with other cells [51, 58]. Harvesting EVs from 2D monolayers was a simple way for us to study EVs from each cell type in isolation and without cross-contamination, but isolation from 3D cultured cells may be required in the future. Here, effects were observed at a single EV dose, requiring future dose optimization studies. Additionally, the applied concentration of CM-EVs was higher than that of EC-EVs. These EV doses were motivated by previous studies [28, 29] and designed to recapitulate, to some extent, the environment in our fibrin hydrogel cardiac tri-cultures. Since CM differentiation cultures (51.3% cTnT+) contained other cell types, one limitation of this study was that CM-EVs were not derived purely from cardiomyocytes, but may have included some EVs secreted by other cell types in the differentiation culture. In the tri-culture system, the number of CMs seeded in hydrogels is five times higher than the number of ECs; therefore, the abundance of CM-EVs is expected to be higher than that of EC-EVs. We narrowed the scope of our study to the direct effects of CM- and EC-EVs on EC vasculogenesis in engineered tissues but note that these EVs also likely influence the physiology of CMs and DPSCs in ways that could indirectly mediate EC vessel formation, necessitating follow-up studies of cell-type specific mechanisms. Identification and validation of specific EC-EV miRNA targets that mediate pro-vasculogenic effects in our tissues will also be the subject of further studies, building on previous work that has already confirmed the role of several miRNA targets that we identified in our predictive analyses here. We further noted from our observation of enhanced cTnT coverage and expression that supplemental EC-EVs may have other benefits in tissue engineering that warrant future studies beyond our focus on vascularization here, such as influences on CM phenotype and function. Though miRNA cargo was at the centre of our investigation as a primary focus of studies in EV biology and therapeutics to date, EV proteins represent another important avenue by which they can modulate recipient cell physiology [12, 44].

## 5. Conclusion

In this study, we demonstrated that CMs disrupt vascular self-assembly in engineered cardiac tissues and that CM-secreted EVs contribute to this effect. We also identified a new potential application for EC-EVs as a supplement for engineered cardiac cultures to enhance vessel formation within *in vitro* tissues. When applied in the right context, EC-EVs and two of their associated key miRNAs, let-7b-5p and miR-126-3p, had both predicted and observed benefits on vascular maintenance and continuity, augmenting pro-vasculogenic signaling in cardiac constructs. By first understanding and then harnessing the power of EV signaling in engineered cardiac constructs, we engineered biofabricated cardiac tissues with microvasculature for potential studies in cardiac physiology, modeling diseases *in vitro*, and screening regenerative therapeutics.

## 6. Methods

### 6.1. Cell culture and differentiation

GFP expressing HUVECs (Angio-Proteomie) were grown in EGM2 culture media (PromoCell) on flasks pre-coated with a 0.2% solution of porcine gelatin (Sigma-Aldrich) dissolved in PBS. DPSCs (Lonza) were grown in DMEM (1 g l^−1^ D-glucose, 110 mg l^−1^ sodium pyruvate) supplemented with 10% FBS, 1% GlutaMAX, 1% Pen-Strep, and 1% NEAA (all from Gibco). iPSC-derived CM were differentiated from the BJ1D human iPSC line (male) according to previously established protocols [17, 59]. Briefly, iPSCs were passaged onto 12-well Matrigel (Corning) coated plates at a density of 0.75 million cells per well and grown in mTeSR Plus medium (STEMCELL Technologies) until fully confluent (up to 48 h). Cardiac induction was initiated by adding RPMI medium supplemented with B27 minus insulin (Gibco) and 1% Pen-Strep, with the addition of 8 *µ*M CHIR99021 (Cayman Chemical) for 24 h. On day 3, 5 *µ*M IWP4 (Stemgent) was added to base media for 48 h. Thereafter, cells were cultured in RPMI containing B27 minus insulin up to day 7 when media was changed to RPMI with B27 supplement (Gibco) for the remainder of culture. CMs were collected after day 14 via treatment with 10X TrypLE Express (Gibco) for up to 30 min at 37 °C followed by neutralization with excess media, centrifugation for 5 min at 300 g, and washing.

### 6.2. Seeding 3D fibrin hydrogel models of vasculogenesis

HUVEC, DPSC, and CM (when appropriate) were seeded in 3D fibrin hydrogels as previously described to model cardiac vasculogenesis *in vitro* [17]. To create EC/DPSC tissues, HUVEC and DPSC monocultures were each treated with 0.05% trypsin (Gibco) for 5 min at 37 °C before neutralization in excess media and centrifugation for 5 min at 300 g. Cells were resuspended for counting and 20 000 HUVEC were mixed with 20 000 DPSCs for each tissue to be seeded. Mixed cells were centrifuged for 5 min at 300 g, media was aspirated, and the cell pellet was resuspended in 7 *µ*l of human fibrinogen (Sigma-Aldrich) stock solution (33 mg ml^−1^ in ddH_2_O containing 20 mM HEPES, 0.9% NaCl) per tissue to be seeded, keeping suspended cells on ice. To seed tissues, 7 *µ*l of cell-fibrinogen suspension was mixed very briefly by pipetting 3-5 times in a microcentrifuge tube containing 2 *µ*l of human thrombin (Sigma-Aldrich) stock solution (25 U ml^−1^ in PBS containing 0.1% BSA). 7 *µ*l of cell-fibrinogen-thrombin mixture was then immediately withdrawn and pipetted onto the middle of a well on a 48 well plate. Upon completion of seeding, 300 *µ*l of media was carefully added to each tissue well, consisting of 60% EGM2 and 40% i3M (complete StemPro-34 (Gibco) supplemented with 20 mM HEPES, 1% GlutaMAX, 1% pen-strep, 0.15 mg ml^−1^ transferrin (Sigma-Aldrich), and 213ug ml^−1^ 2-phosphate ascorbic acid (Sigma-Aldrich)). EC/DPSC/CM tissues were created using an identical procedure with the addition of 100 000 iPS-CM per tissue to be seeded added to the initial mixed cell suspension. Tissues were incubated at 37 °C and 5% CO_2_, media was changed every two days. For EV uptake assessment via flow cytometry, fibrin plugs were seeded with non-GFP HUVEC. To test the effects of different cell ratios/densities, fibrin tissues were also seeded using the same protocol above but with 60 000 HUVECs/60 000 DPSCs, or 20 000 HUVECs/100 000 DPSCs.

### 6.3. Tissue imaging and immunostaining

Tissues were imaged in the green fluorescent channel (FITC, Olympus IX81 fluorescent microscope) every two days to track organization of GFP+ HUVEC into self-assembled vessels. On day 10, tissues were washed gently with PBS, fixed in 4% formaldehyde (ThermoScientific) for 1 h at room temperature in the dark, then washed three times with PBS and blocked overnight at 4 °C in PBS containing 10% FBS and 0.1% TRITON-X (Cayman Chemical) for immunostaining as previously described [17, 28]. Briefly, tissues were incubated with mouse monoclonal anti-Troponin-T (1:200, ThermoScientific MA512960) or rabbit polyclonal anti-VE Cadherin (1:400, Abcam ab33168) overnight at 4 °C. Tissues were washed 3 × 15 min in PBS followed by secondary incubation using AF647-conjugated goat anti-mouse antibody (1:400, ThermoScientific A-21240) or AF647-conjugated donkey anti-rabbit antibody (1:400, ThermoScientific A-32795) overnight at 4 °C. For cTnT quantification, blocked and permeabilized tissues were directly stained with APC-conjugated anti-cTnT (1:100, Miltenyi Biotec 130-120-403). Following staining, all tissues were washed 3 × 15 min in PBS, stained with DAPI (1:1000, Sigma-Aldrich), washed, then imaged on a Nikon A1R confocal microscope.

### 6.4. Image analysis

For PI quantification, 10x images were analyzed using ImageJ [60]. Thresholding was performed on the DAPI channel, followed by binary processing (erode, watershed, and fill holes) and cell counting using the ‘analyze particle’ function (size 2—infinity). Thresholding was also used on the PI channel. The ‘image calculator’ function was used to select overlapping pixels between DAPI and PI channels (PI positive nuceli) followed by particle counting (size 0—infinity). The proportion of dead cells was calculated (PI positive nuclei/total nuclei) and averaged among three regions of the same sample for each biological replicate.

For cTnT quantification, 10x images were analyzed using ImageJ [60]. DAPI thresholding and cell counting was performed as before. Raw, unedited images in the cTnT channel were analyzed using the ‘measure’ function to record % area of cTnT+ pixels as well as raw integrated density of cTnT staining. Both metrics were normalized to the number of nuclei counted for a given image. The results for 3 different regions of a given sample were averaged for each individual biological replicate. cTnT eccentricity was calculated using a custom Matlab code as described previously to assess the degree of CM ellipticity/elongation [17].

### 6.5. Quantification of vasculogenesis

Fluorescent microscope images of GFP+ HUVEC within tissues were obtained as described at a magnification of 10x using a consistent exposure time and laser power, focusing on a vertical plane in the middle of each tissue to have the majority of cells in focus for a given frame. Images were loaded in ImageJ [60] to automatically adjust brightness and convert file type to jpg. Angiotool software [61] was used to quantify average and total vessel length, average lacunarity, total number of junctions and total number of endpoints across replicates for each sample group. Vasculature quantification was collected at an intensity scale of 12–255 for each image, which was optimized to include as many faint vessels as possible while removing non-specific high-intensity pixels. Small non-vasculature particles in the foreground and background of the images were disregarded from the measurements using the software’s automatic noise filter. Dark spots and holes within the vasculature were also eliminated using the ‘fill holes’ filter.

Average vessel width was measured on ImageJ [60] using the same images from Angiotool analyses described above. Five location points on each image with consistent, pre-defined coordinates were highlighted and the width of the closest in-focus, elongated vessel to each point was measured at its widest point before the start of a new branch. The widths measured at all five pre-defined locations were averaged for a given image. Where fewer than five elongated vessles/ECs were observed in a given image, the average was taken for as many elongated vessels as existed, excluding round ECs.

### 6.6. Viability and metabolic analysis of tissues

Conditioned media from EC/DPSC and EC/DPSC/CM tissues was collected every two days during regular media changes and frozen. The LDH cytotoxicity assay kit (Cayman Chemical) was used to quantify LDH concentration in conditioned media from each day of culture and group according to the manufacturers protocol. After removal of conditioned media every two days, alamar blue cell viability reagent (Invitrogen) was used to measure the relative metabolic activity of tissues. 30 *µ*l of alamar blue reagent was mixed with 280 *µ*l of culture medium and applied to each tissue well to be characterized. Tissues were incubated at 37 °C and 5% CO_2_ for 4 h prior to collection of conditioned alamar blue-containing medium and returning to regular maintenance medium. 100 *µ*l of conditioned alamar blue medium from each sample was added to separate wells on a 96 well plate and fluorescence intensity was measured on a SpectraMax i3 (Molecular Devices) plate reader at excitation and emission wavelengths of 560 and 590 nm respectively. After day 10 collection of media, tissues were washed with PBS. Propidium iodide (PI; Invitrogen, 1.0 mg ml^−1^ ) was diluted with PBS (75 *µ*l per ml PBS) and 300 *µ*l of diluted PI stain was added to each tissue well to be stained prior to 30 min of incubation at 37 °C. Tissues were washed, fixed, stained with DAPI, and imaged on a confocal microscope as described previously.

### 6.7. Flow cytometry

Fibrin hydrogels were digested on day 8 of culture via incubation in 300 *µ*l of collagenase type 2 (200 units ml^−1^, Worthington LS004205) supplemented with DNase I (10 *µ*g ml^−1^, Millipore Sigma 260 913-10MU) and ROCK inhibitor (10 *µ*M, Biorbyt orb154626) for 1 h at 37 °C, with agitation via pipetting every 15 min. After 1 h, 300 *µ*l of 10x TrypLE Select (ThermoFisher Scientific A1217701) was added, followed by pipetting to disrupt remaining aggregates and quenching with 500 *µ*l of culture media. For EV uptake assessment, each individual fibrin tissue was assessed as a single biological replicate. For other flow analyses, two digested fibrin tissues were pooled together for assessment as a single biological replicate. Dissociated cells were centrifuged for 5 min at 300 g then fixed in 4% formaldehyde for 15 min at room temperature. Fixed cells were diluted with 9 ml PBS, centrifuged again, then suspended in blocking buffer (10% FBS and 10 *µ*g ml^−1^ DNase in PBS) for 30 min at room temperature. Following blocking, cells were centrifuged and resuspended in permeabilization buffer (5% FBS, 10 *µ*g ml^−1^ DNase, 0.25% Triton-X in PBS) then stained with APC-conjugated cardiac troponin T antibody for 1 h (where appropriate; 1:100 in permeabilization buffer; Miltenyi Biotec 130-120-403). Finally, cells were centrifuged and washed once with FACS buffer (5% FBS, 10 *µ*g ml^−1^ DNase in PBS) before resuspension in 500 *µ*l FACS buffer. Cells from digested tissues were analyzed via flow cytometry (Cytek Aurora (violet (405 nm), blue (488 nm), and red laser (640 nm)), Fremont, California, USA). To identify single cells, all events were first gated by forward scatter area (FSC-A) versus side scatter area (SSC-A). FSC-A versus forward scatter height (FSC-H) was then used for doublet exclusion. GFP+ or cTnT+ events were identified based on count (% of mode) versus fluorescent intensity with respect to identically digested tissues containing unstained, non-GFP cells. Flow cytometry results were analyzed in FlowJo^TM^ v10.10 Software (BD life sciences) [62]. cTnT expression in iPS-CM differentiation culture was assessed using a similar preparation and flow cytometry protocol, with the inclusion of isotype control (1:50, Miltenyi Biotech 130-118-354) and using FITC-conjugated cTnT antibody (1:50, Miltenyi Biotech 130-119-575).

### 6.8. Thin layer fibrin hydrogel seeding

ECs, DPSCs, and CMs were cultured and harvested as described earlier, then suspended in fibrinogen at the same concentration and cell densities previously used for 3D fibrin hydrogels (including both EC/DPSC and EC/DPSC/CM groups) but this time scaled to a volume of 11 *µ*l of fibrinogen per tissue to be seeded. 11 *µ*l of cells/gel was withdrawn and mixed briefly with 3.14 *µ*l of thrombin prior to seeding 11 *µ*l of cell/gel mixture into one well of a 48-well plate. Immediately after seeding, the empty sterile pipette tip was used to ‘paint’ the hydrogel droplet around the well surface quickly before hydrogel cross-linking, dispersing the droplet to form a thin layer of hydrogel that uniformly coated the well surface (without wicking up the well edge). Media changes, imaging, and vasculature analyses were performed as described previously.

### 6.9. ELISA analysis of conditioned media

Conditioned media collected from EC/DPSC and EC/DPSC/CM tissues on day 10 was analyzed by ELISA (Simple Plex Ella platform, ProteinSimple) to quantify concentrations of endothelin-1, IL-6, IL-8, TREM-1, E-selectin, ICAM-1, and VCAM-1 according to the manufacturer’s protocol and as previously described [63]. A human angiogenesis array (RayBiotech) was used to quantify concentrations of Angiogenin, VEGF, HGF, PDGF, EGF, ANGPT-2, PIGF, bFGF, HB-EGF, and leptin according to the manufacturer’s protocol. A GenePix^®^ Professional 4200 Microarray Scanner was used to measure fluorescence at 530 nm [28].

### 6.10. Proteomic analysis of secretome

Proteomic analysis of EC/DPSC and EC/DPSC/CM secretome was performed according to a previously described protocol [17]. Briefly, EC/DPSC and EC/DPSC/CM tissue media was changed to phenol red-free media on day 8 and conditioned media was harvested from samples for both groups on day 10, pooling media from 3 tissues together for each singular biological replicate. Cell debris was removed via centrifugation for 20 min at 2500 g and 4 °C. Amicon 3kDa filters (Millipore) were used to concentrate the supernatant from each sample by centrifugation for 1 h at 4000 g and 4 °C. Concentrate was dried under medium heat for two hours using a SpeedVac device (ThermoFisher Scientific). For each sample, 20 *μ*g of dried protein was denatured and reduced (100 mM Tris-HCl, pH 8.5, containing 8 M urea, 10 mM DTT) at 60 °C for 30 min. 100 mM iodoacetamide was applied for 15 min to alkylate samples before quenching with 40 mM DTT and dilution with 50 mM ammonium bicarbonate solution to achieve a urea concentration below 1 M. 0.2 *μ*g of trypsin (Sigma-Aldrich) was applied overnight at 37 °C to digest proteins followed by acidification and de-salting using custom C18 pipette tips.

LC-MS/MS was performed using an Easy nLC-1200 system connected to a ThermoQExactive HF mass spectrometer (ThermoFisher Scientific) using a Top 20 data-dependent acquisition mode. Buffer A (0.1% formic acid) and Buffer B (0.1% formic acid in 80% acetonitrile) constituted the mobile phase. Peptides were eluted through a PepMap RSLC C18 column (2 *μ*m, 75 *μ*m × 50 cm) and a PepMap 100 C18 precolumn (3 *μ*m, 75 *μ*m × 2 cm), employing a 120 minute gradient of Buffer B from 5% to 40%. MaxQuant software (v1.6.10.43) [64] was used for data processing, employing an FDR threshold of 0.01 and default label-free quantification (LFQ). Downstream PCA, differential, and network analyses were performed using Perseus software (v1.6.5.0) [65] and the Cytoscape StringApp [66]. GO analysis was performed using the PANTHER (v18.0) [67, 68] statistical overrepresentation test, sorting terms by FDR. The mass spectrometry proteomics data have been deposited to the ProteomeXchange Consortium via the PRIDE [69] partner repository with the dataset identifier PXD047960.

### 6.11. EV isolation and NTA

HUVECs (Angio-Proteomie) grown in 2D monolayers as described above were cultured until reaching 80% confluence. Cells were then washed with PBS and media was changed to basal EGM2 supplemented only with 2% exosome-depleted FBS (Gibco). Conditioned media was collected after 48 h and frozen at −20 °C for storage. Conditioned media was also directly collected from CMs (due to absence of serum in media) after 48–72 h of culture and between days 16-20 of differentiation (as described above) prior to storage at −20 °C. To isolate EVs, conditioned media from EC and CM was thawed and centrifuged at 3200 g for 10 min at 21 °C to pellet large debris. Supernatant was collected and mixed with 0.4 volumes of 0.2 *μ*m filter sterilized buffer containing 28% w/v PEG 6000 (Sigma-Aldrich) and 1.75 M NaCl (Sigma-Aldrich) to reach a final concentration of 8% PEG and 0.5 M NaCl as optimized previously [70]. PEG-media mixtures were rotated gently overnight at 4 °C prior to centrifugation at 3200 g for 30 min at 21 °C to pellet EVs. Supernatant was discarded, pellets were gently rinsed with PBS, then PBS was aspirated without disturbing pellets.

EV pellets were resuspended in exosome resuspension buffer (Qiagen) to create a 100x concentrated EV solution based on the initial volume of conditioned media from which they were isolated. Aliquots of concentrated EVs were diluted to 1x in PBS for NTA characterization of size distribution and concentration using a Nanosight NS300 (Malvern). Concentrated EVs were stored at −80 °C.

### 6.12. EV Western Blots

Pelleted EVs were directly resuspended in 120 *μ*l ice-cold RIPA buffer (ThermoFisher), performing several minutes of alternating cycles of vigorous pipetting and brief vortexing to lyse EVs. Lysed EVs were kept on ice or frozen at −20 °C until use. Protein concentration was quantified using the Pierce BCA Protein Assay kit (ThermoFisher Scientific) according to the manufacturer’s protocol. To perform western blots, 10 *µ*g of protein solution was mixed with 4x Sample Loading Buffer (LI-COR) and 10x BOLT Sample Reducing Agent (ThermoFisher Scientific), diluted to 40 *µ*l of volume with DI water, and incubated for 10 min at 70 °C. For non-reducing blots (to detect CD63), no reducing agent was added and samples were incubated at room temperature for 20 min. Samples were briefly centrifuged and loaded into Bolt 4%–12% Bis-Tris Gels (Invitrogen). Electrophoresis was run at 200 V (max. 120 mA) for 35 min (or until protein front reached the bottom of gels) in Bolt MOPS SDS Running Buffer (Invitrogen). DI water was used to rinse gels. Transfer to a methanol-activated Immobilon-FL PVDF membrane (Millipore Sigma) was performed using the iBlot Gel Transfer Device (Invitrogen) at 20–25 V for 7 min.

REVERT Total Protein Stain (LI-COR) was used according to the manufacturers protocol, imaging on a LI-COR Odyssey Fc Imager (LI-COR). 5% skim milk buffer was used for 1 h of blocking at room temperature. Primary staining was performed overnight at 4 °C under gentle rotation using mouse anti-CD63 (Abcam, ab271286, 1:1000; non-reducing blots) or rabbit anti-ALIX (ThermoFisher Scientific, MA5-32773, 1:1000; reducing blots) in skim milk buffer with 0.2% tween-20. Blots were washed 3x5min in TBS + 0.2% tween-20 (TBST), then incubated in skim milk buffer containing 0.2% tween-20, 0.01% SDS, and either anti-mouse IRDye800CW secondary antibody (LI-COR, 925-32212, 1:10,000) or anti-rabbit IRDye800CW (LI-COR, 925-32213, 1:10000). After 2 h of incubation at room temperature in the dark, blots were washed 3 × 5 min in TBST, rinsed in TBS, then imaged for 10 min on the 800 nm channel of a LI-COR Odyssey Fc.

### 6.13. TEM

Carbon-coated copper TEM grids (Electron Microscopy Sciences) were plasma treated and 5 *μ*l of 100x EV concentrate was deposited onto each grid for 1 min before wicking with filter paper. Grids were washed by pipetting and then wicking 5 *μ*l of DI water. 5 *µ*l 2% uranyl acetate solution (Electron Microscopy Sciences) was pipetted onto each grid for 30 s followed by wicking. A Talos L120C TEM (ThermoFisher Scientific) was used to acquire images of EVs at 45,000x magnification.

### 6.14. EV miRNA sequencing

Pelleted EVs were lysed for miRNA isolation using the miRNeasy Micro Kit (Qiagen) according to the manufacturers protocol. For CM-EVs, miRNA enrichment and DNase treatment steps from the standard protocol were performed in-house prior to Qubit (ThermoFisher Scientific) and Agilent 2100 Bioanalyzer (Agilent) quantification at the Donnelly Sequencing Centre (Toronto, ON). The SEQuoia Complete Stranded RNA Library Prep Kit and Dual Indexed Primers (Bio-Rad) were used to prepare miRNA libraries for sequencing using a NextSeq500 (Illumina) at a read length of 1x75bp and an average depth of 30 million reads (Donnelly Sequencing Centre, Toronto, ON). Reads were aligned to the human genome (GRCh38.p12) via Bio-Rad SeqSense employing STAR aligner (v2.7.0 f) to generate miRNA count files. For EC-EVs, isolated total RNA was sent to Novogene for DNase treatment and sequencing as previously described [28]. After Qubit and Bioanalyzer quantification, the QIAseq miRNA Library Kit (Qiagen) was used to prepare libraries for sequencing on a NovaSeq S4 (Illumina) at 2x150bp and an average depth of 12 million reads. R1 reads were trimmed to 75bp and aligned to the human genome (GRCh38.103) via the miRbase database (v22) using the Qiagen RNA-seq Analysis Portal 3.0 (workflow version 1.2) to generate miRNA count files.

### 6.15. Downstream analysis of EV-miRNA

PCA was performed on raw miRNA count files using PCAGO [71]. DESeq2 was used for differential expression analysis via the Galaxy platform [72, 73]. For both CM- and EC-EV miRNA, a cutoff of upregulated miRNA within the top 20 most abundantly expressed species was used to select key miRNA for further pathway analyses. Key miRNA from both EV types were used in separate KEGG pathways union analyses via DIANA-mirPath (v.3) [74] employing the microT-CDS database (v5.0) for target prediction using a cutoff of P < 0.05, target score threshold of 0.8, then FDR correction of P values. Pathways with FDR < 0.05 for either or both EV types were selected for plotting. For selected pathways, associated P-values of individual key miRNAs were tabulated from mir-Path results (increasing cutoff to P < 1 to find results for pathways significant in one EV species but not the other) and plotted on a heatmap using Morpheus by Broad Institute (RRID:SCR_017386) applying default hierarchical clustering of rows.

Key miRNA target prediction was also performed on miRDB [75, 76] using a cutoff target score of 80. GO CC, BP, and MF analysis of predicted targets was performed on PANTHER as described earlier, with pathways sorted by either fold enrichment or FDR [67, 68]. Results were plotted using SRPlot (www.bioinformatics.com.cn/srplot).

### 6.16. HUVEC scratch-wound assay and Ki-67 staining

Two-well culture inserts (Ibidi) were placed in wells of a 24 well plate. 70 *μ*l of trypsinzed and counted GFP+ HUVEC cell suspension (0.33 million cells/ml in EGM2) was seeded into both wells of each insert. After 24 h, inserts were removed and wells of 24 well plate were each filled with 500 *μ*l of EGM2 containing either EC-EVs (5 *μ*l/ml, ∼6.3x10^7^ EVs/ml), CM-EVs (100 *μ*l ml^−1^, ∼1.0 × 10^9^ EVs ml^−1^), or no EVs. Fluorescent imaging of the green channel as described before (4x) was used to visualize gap closure at 0, 2, 4, 6, 8, and 24 h. After cropping to isolate and center gap in frame and identical brightness adjustment for all images, quantification of gap closure was performed using the ImageJ Wound Healing Size Tool [77] plugin only changing the following settings: variance window radius of 35; threshold of 254; percentage of saturated pixels 0.4.

Cells were washed and fixed as before, permeabilized with 0.3% triton-x for 15 min, washed 3 times with TBS, then blocked with 5% normal goat serum (NGS, ThermoFisher Scientific) for 1 h at room temperature in the dark with gentle shaking. Cells were treated with AF-647 conjugated rabbit anti-Ki-67 (Cell Signaling Technology, 12075S, 1:50 in 5% NGS) then DAPI as before (1:2000). Stained cells were imaged (10x) on a fluorescent microscope as before in the green, blue, and far-red channels. Quantification was performed on ImageJ by thresholding to distinguish Ki-67 positive nuclei from background, binary processing to fill gaps between positive signals within the same nuclei, then counting using the analyze particles feature followed by normalization to total number of DAPI positive nuclei.

### 6.17. EV treatment of fibrin tissues

Seeding and culture of EC/DPSC and EC/DPSC/CM hydrogels was performed as before. Isolated CM-EVs (100 *μ*l ml^−1^, ∼1.0 × 10^9^ EVs ml^−1^) were directly added to the culture media of a subset of EC/DPSC tissues right after seeding and for each subsequent media change every 48 h until day 10. The same procedure was followed for the supplementation of EC-EVs (10 *μ*l ml^−1^, ∼1.3 × 10^8^ EVs ml^−1^) to a subset of EC/DPSC/CM tissues. Tissue imaging, quantification of vascular tube formation, and immunostaining of VE-Cadherin and cTnT was performed as described earlier.

### 6.18. EV labelling and uptake

EC-EVs were labelled with lypophilic dye and fluorescent imaging was used to verify EV uptake by HUVEC cells in 2D as previously described [28, 78]. Briefly, 100x concentrated EC-EV suspension was mixed at a 1:3 ratio with diluted CellTracker CM-DiI Dye (Invitrogen C7001; 1 mg ml^−1^ in DMSO, diluted to 5 *µ*g ml^−1^ in PBS) for 15 min at room temperature. Dyed EVs were concentrated in an Amicon Ultra-0.5 ml 100kDa filter unit (MilliporeSigma) by centrifugation for 5 min at 14 000 g. Dyed EV suspension was washed twice by successive additions of 500 *µ*l of PBS to the filter unit, gentle pipetting, and centrifugation for 5 min at 14 000 g. EVs were collected in a fresh tube by inverting the filter units and centrifuging for 2 min at 1,000 g. Washed and dyed EVs were added to the culture media (∼0.5 × 10^9^ EVs total) of GFP+ HUVEC (80% confluent) for 2 h. HUVEC were then washed twice with PBS, fixed, stained with DAPI as described earlier, and imaged on a confocal fluorescent microscope (Nikon A1R). Stained EVs were also assessed by flow cytometry as described earlier to assess concentration loss from staining and to confirm the presence of fluorescent signal.

For the 3D EV uptake assay and flow cytometry, EC/DPSC/CM fibrin hydrogels were seeded as described earlier but this time using non-GFP HUVECs. EC-EV supplementation was performed as before. On day 4 of culture (the midpoint), stained EVs prepared as above were supplemented to fibrin tissue culture media at a scaled-up volumetric concentration to account for EV loss during staining. 80 *µ*l of EC-EVs were stained per ml of supplemented culture media required, and the final volume of stained EVs (which was consistently approximately 27 *µ*l in Amicon filters after staining and washing steps) was then diluted into an appropriate volume of media (0.3 ml per tissue to be treated) before addition to fibrin tissues. After 24 h of culture, tissues were digested and analyzed via flow cytometry as described previously on a BD FACSAria Fusion (violet (405 nm), blue (488 nm), yellow-green (561 nm), and red (640 nm)) flow cytometer using the 561 nm laser. Gating was performed as described earlier to select cells, single cells, then DiI+ cells as compared to tissues treated with unstained EVs. Stained and unstained EVs were also assessed by flow at 1:10 dilution (based on original volume of 100x concentrated EV preparations) in 0.2 *µ*m filtered PBS.

### 6.19. miRNA transfection

hsa-let-7b-5p (ThermoFisher Scientific 4464 066, Assay ID MC11050), hsa-miR-126-3p (ThermoFisher Scientific 4464 066, Assay ID MC12841), and miRNA mimic negative control (ThermoFisher Scientific 4464058) were suspended in nuclease-free water at a concentration of 50 *µ*M. HUVECs and DPSCs were trypsinized and separately pre-plated onto 12 well plates at a density of 50 000 cells well^−1^. Differentiated iPS-CMs were dissociated and plated on a 0.2% gelatin-coated 48-well plate at a density of 185 000 cells well^−1^. Cells were cultured for 24 h prior to transfection. Transfection was performed as detailed below at a miRNA concentration of 30 nM based on prior published work and manufacturer recommendations [49, 79].

For 12-well plate transfections (ECs, DPSCs), 0.6 *µ*l of each miRNA mimic was separately mixed with 25 *µ*l Opti-MEM media (Gibco 31985070) per well to be transfected. In separate vials, aliquots of 0.6 *µ*l of lipofectamine 2000 (ThermoFisher Scientific 11668019) were also mixed with 25 *µ*l Opti-MEM media. After 5 min of incubation, equal volumes of miRNA and lipofectamine preparations were mixed (in separate tubes for each mimic) and incubated for 20 min for miRNA encapsulation. For each of the 3 individual miRNA mimics, 50 *µ*l of lipofectamine encapsulated miRNA was separately added to the media (1 ml) of individual 2D monocultures of ECs and DPSCs to transfect cells for 24 h at 30 nM. For 48-well plate transfections of CMs, the same procedure was followed (scaling all volumes down by a factor of 5 to account for the smaller well size).

After 24 h of transfection, cells were washed with PBS, dissociated, counted, and seeded into 3D fibrin hydrogels using the same procedure as described earlier. Separate groups of tissues were made from cells (ECs, DPSCs, and CMs) that were transfected with let-7b-5p, miR-126-3p, or miRNA mimic negative control. Tissues were cultured, imaged, and analyzed using the procedures previously described.

### 6.20. Transfection efficiency control

2D transfection efficiency of ECs, DPSCs, and CMs was assessed by replating cultures of each type of cell separately at a density of 20 000 cells well^−1^ of a 96-well plate for 24 h. Separate mixtures of 1:50 TYE DS 563 Transfection Control fluorescent DsiRNA (Integrated DNA Technologies 51-01-20-19) in Opti-MEM and 1:50 lipofectamine 2000 in Opti-MEM were incubated for 5 min separately, then mixed in equal ratios for 20 min to encapsulate siRNA. Encapsulated fluorescent siRNA was added (1:20) to the media of each cell type for 24 h. Cells were then washed, fixed, DAPI stained, and imaged at 20x on an Olympus fluorescent microscope as described earlier. DAPI thresholding, binary processing, and cell counting was also performed as described. DAPI images were overlayed with the fluorescent siRNA (red) channel for manual counting of cells that were positively associated with siRNA. Percentage of siRNA transfected cells (nuclei associated with siRNA/ total number of nuclei) was calculated and averaged between three regions of the same sample for each individual biological replicate.

### 6.21. Fabrication of vascularized cardiac tissue-on-a-chip models

Angiotubes were fabricated and assembled onto the InVADE platform as described previously [5, 6, 80]. Briefly, poly(octamethylene maleate (anhydride) citrate) (POMaC) pre-polymer was synthesized according to a defined protocol [80]. Master molds for Angiotubes (separate top and bottom molds) and plate bases were prepared via soft lithography followed by the creation of PDMS negative templates [6]. Plate bases were prepared via hot embossing of polystyrene sheets on the PDMS template. Angiotube templates were perfused with POMaC pre-polymer then exposed to UV light for partial cross-linking. Top and bottom layers of tubes were joined via 3D stamping followed by a final UV cross-linking step. After manual placement of Angiotubes on polystyrene base plates, polyurethane glue was used to bond a 96 square well upper-structure to bases. Plates were sterilized with 0.2 *µ*m filtered 70% ethanol for 2 h followed by PBS washing, coating in 0.2% gelatin for 2 h, and soaking in culture media overnight. EC/DPSC/CM fibrin hydrogels were seeded in the middle of tissue wells on top of tubes using the same procedure, volumes, and concentrations as described earlier. EC-EV supplementation was performed at the same frequency and concentration as described. Plates were placed on a rocker in an incubator set to a 20 degree angle and alternating tilt at 0.1 cycles/min. Images and videos were captured on an Olympus fluorescent microscope on day 6 after cardiomyocytes started to contract. idenTx9 plates (AIM Biotech IDTX9) [31] were obtained and seeded with cells at a similar density as before (scaled to the increased volume of tissue wells) using a protocol previously optimized for seeding a similar microfluidic vascularized chip [7]. For each chip to be seeded, 29 000 HUVECs, 29 000 DPSCs, and 143 000 CMs were suspended in 11.5 *µ*l fibrinogen stock solution (diluted to 10 mg ml^‒1^ with PBS), which was then added to 2.3 *µ*l of thrombin stock solution (diluted to 1 U ml^‒1^ with PBS). This mixture was then withdrawn using a P20 pipette. The pipette tip was placed in one of the tissue well inlet ports and cell/gel solution was partially released very slowly until the gel front reached the middle of the tissue well. The pipette tip was then withdrawn and placed in the inlet port on the opposite side of the chip, where the remainder of cell/gel solution was gently added only until the two gel fronts met in the middle of the tissue well without causing spill-over into media channels. PBS was added to reservoirs beside chips to maintain humidity and tissues were allowed to gel for 30 min at 37 °C. Media was supplemented with 20 *µ*g ml^−1^ aprotinin (Sigma-Aldrich A3428-25 mg) before addition of 50 *µ*l media to the left port and 70 *µ*l to the right port for both the top and bottom media channels. EC-EVs were supplemented to media at the same concentration as before. Media was changed daily, and tissues were imaged as before after an additional 48 h of culture.

### 6.22. Data and statistical analysis

Statistical analysis and plotting, unless otherwise indicated, were performed using GraphPad Prism (v10.1.0). Data are presented as mean ± standard deviation (SD) with sample sizes indicated in each figure. Where data passed the Shapiro–Wilk normality test, differences between groups were analyzed by student t-test with post-hoc Holm–Šídák test for multiple comparisons, repeated measures two-way ANOVA with Geisser–Greenhouse correction followed by Tukey’s multiple comparisons with individual variances computed for each comparison, or Welch’s t-test as indicated and appropriate. When data did not pass the Shapiro–Wilk test, the non-parametric Mann–Whitney Test with post-hoc Holm–Šídák test for multiple comparisons was applied as indicated. The Prism ROUT test was used to identify outliers (using a cut-off of *Q* = 1%) where appropriate, with one statistical outlier sample found and removed in figures 2(e) and 7(i) (EC/DPSC day 8). The Grubbs outlier test was used to find individual outliers (alpha = 0.05) where appropriate, with one statistical outlier found and removed in figures 9(b)–(d) (EC/DPSC/CM). *P* < 0.05 or FDR-adjusted *P* < 0.05 (where appropriate) was considered statistically significant.

## Data Availability

All data that support the findings of this study are included within the article (and any supplementary files).
